# The Silent Link: Exploring the Impact of Periodontal Diseases on Head and Neck Carcinogenesis

**DOI:** 10.1002/cre2.70341

**Published:** 2026-05-03

**Authors:** Yashmin Afshar, Nima Rezaei

**Affiliations:** ^1^ Cancer Immunology Project (CIP) Universal Scientific Education and Research Network (USERN) Tehran Iran; ^2^ School of Medicine Tehran University of Medical Sciences Tehran Iran; ^3^ Research Center for Immunodeficiencies, Children's Medical Center Tehran University of Medical Sciences Tehran Iran; ^4^ Network of Immunity in Infection, Malignancy and Autoimmunity (NIIMA) Universal Scientific Education and Research Network (USERN) Tehran Iran; ^5^ Department of Immunology, School of Medicine Tehran University of Medical Sciences Tehran Iran

**Keywords:** head and neck cancer, oral microbiome, periodontitis

## Abstract

**Objectives:**

Oral dysbiosis can accelerate the progression of head and neck squamous cell carcinoma (HNSCC) by fostering a pro‐inflammatory, immunosuppressive, and metabolically altered environment. This narrative review examines the relationships between periodontitis‐associated bacteria and HNSCC, focusing on their impact on oncogenic pathways, immune modulation, and epigenetic alterations.

**Materials and Methods:**

A comprehensive search of PubMed and Google Scholar was conducted up to January 28, 2026, without time limitations, using all relevant keywords related to HNSCC, head and neck cancers, periodontitis, and the oral microbiome.

**Results:**

Key periodontitis‐associated bacteria, including *Fusobacterium nucleatum*, *Porphyromonas gingivalis*, *Capnocytophaga gingivalis*, and *Prevotella intermedia*, may play a vital role in HNSCC. These bacteria stimulate several oncogenic pathways, including Wnt/β‐catenin, NF‐κB, and PI3K/Akt, enabling HNSCC to evade immune responses, trigger epithelial‐to‐mesenchymal transition and angiogenesis, and encourage cell proliferation and stemness. Furthermore, microbial interactions within the tumor microenvironment significantly impact treatment resistance, particularly in the context of immune checkpoint inhibitor therapy.

**Conclusions:**

Incorporating periodontal screening, microbiome profiling, and bacterial‐targeted therapies into oncology could enhance treatment outcomes for HNSCC. Future research should investigate CRISPR‐based microbial interventions, targeted epigenetic therapies, and microbiome‐driven precision oncology strategies.

**Abbreviations**
16S rRNA: 16S ribosomal RNA
ACC1: acetyl‐CoA carboxylase 1
AP‐1: activator protein 1
ATR: Ataxia Telangiectasia and Rad3‐Related
Cas: CRISPR‐associated (systems/proteins)
CHK1: checkpoint kinase 1
CNAs: copy number alterations
COX‐2: cyclooxygenase‐2
CRISPR: clustered regularly interspaced short palindromic repeats
CXCL2: C‐X‐C motif chemokine ligand 2
DNMT: DNA methyltransferase
EBV: Epstein–Barr virus
ECM: extracellular matrix
EMT: epithelial‐to‐mesenchymal transition
FAK: focal adhesion kinase
FASN: fatty acid synthase
FISH: fluorescence in situ hybridization
HDAC: histone deacetylase
HNSCC: head and neck squamous cell carcinoma
HPV: human papillomavirus
ICI: immune checkpoint inhibitor
IFN: interferon
IL: interleukin
ISG15: interferon‐stimulated gene 15
JAK: Janus Kinase
LPS: lipopolysaccharide
miRNA: microRNA
NDK: nucleoside diphosphate kinase
NF‐κB: nuclear factor kappa‐light‐chain‐enhancer of activated B cells
NK: natural killer (cells)
NLRP3: NOD‐like receptor family, pyrin domain containing 3
OMVs: outer membrane vesicles
OSCC: oral squamous cell carcinoma
PAMPs: pathogen‐associated molecular patterns
PDCD4: programmed cell death 4
PD‐L1: programmed death‐ligand 1
PI3K: phosphoinositide 3‐kinase
PRRs: pattern recognition receptors
ROS: reactive oxygen species
rRNA: ribosomal RNA
STAT: signal transducer and activator of transcription
TAM: tumor‐associated macrophages
TLR: toll‐like receptor
TNF‐α: tumor necrosis factor alpha
YAP: yes‐associated protein

## 1 Introduction

Head and neck squamous cell carcinoma (HNSCC) is one of the most common cancers, with a 50% mortality rate affecting approximately 900,000 individuals annually. They most frequently emerge in the oral cavity, hypopharynx, nasopharynx, larynx, and oropharynx (Barsouk et al. 2023). Specific risk factors are associated with different histological subtypes of HNSCC. Tobacco consumption and Epstein–Barr virus (EBV) infection are risk factors for nasopharyngeal cancers (Chen et al. 2019). Meanwhile, tumors in the oral cavity, hypopharynx, and larynx are most affected by carcinogens, including tobacco consumption, alcohol misuse, and oropharyngeal SCC occurrence associated with Human papillomavirus (HPV) (Ang et al. 2010; Leemans et al. 2018; Gillison et al. 2000). Each group of carcinogen‐induced HNSCC, EBV + HNSCC, and HPV + HNSCC is recognized as a distinct subclass of HNSCC, as they have different etiologies, molecular biology, clinical behavior, and prognoses. HNSCCs generally endure high genomic instability, with a mean of 141 copy number alterations (CNAs), including both amplification and deletion. HPV^‐^ HNSCCs typically harbor multiple CNAs, including *CDKN2A*, *TP53*, *FAT1*, and *NOTCH1* (Smeets et al. 2009). Recently, a group identified a subgroup of HPV^‐^ HNSCCs with uncommon CNAs, characterized by more frequent mutations in *CASP8, HRAS, PIK3CA, FBXW7, TERT, RAC1, NOTCH1*, and *B2M*, and less frequent mutations in *TP53* and *CDKN2A* (Muijlwijk et al. 2024). This group claims that HPV^‐^ HNSCC with infrequent CNAs is another distinct subclass of HNSCCs, as it has unique genetic mutations, predominantly occurring in older women, and is associated with a better prognosis. Moreover, histologically, they are well differentiated (Muijlwijk et al. 2024). Individuals with carcinogen‐induced HNSCCs experience *TP53* loss of function, CNAs in 3q26/28 and 11q13/22, and mutations in *CDKN2A* (Forbes et al. 2008). HPV^+^ HNSCCs exhibit helical domain mutations in *PIK3CA*, loss of *TRAF3*, and amplification of *E2F1*, while rarely harboring mutations in *CDKN2A* and *TP53*, leading to a significantly better prognosis (Lawrence et al. 2015). Besides HPV and EBV, microbiome shifting (dysbiosis), though not constituting a distinct subclass of HNSCCs, can strongly affect disease outcome by shaping the tumor microenvironment (TME) of HNSCCs, potentially influencing oncogenic pathways and gene expression profiles (Saikia et al. 2023). While genetic alterations drive cancer, the TME, influenced by the microbiome, critically modulates tumor growth, treatment response, and outcomes. Several datasets indicate shifts in the oral microbiome in HNSCC, although no causative relationship was detected; correlations among them are likely. Higher amounts of *Streptococcus spp., Capnocytophaga gingivalis (C. gingivalis), Prevotella melaninogenica, Porphyromonas gingivalis (P. gingivalis), Peptostreptococcus stomatis, Gemella, Johnsonella ignava, Lactobacillus*, and *Prevotella spp* were detected in the HNSCC tissues (Orlandi et al. 2019). Moreover, chronic restraint stress has been shown to promote HNSCCs by altering the oral microbiota. It elevates *Pseudomonas* and *Veillonella* while depleting *Corynebacterium* and *Staphylococcus* species. This host metabolome shift, characterized by increased plasma kynurenine (Kyn), activates the aryl hydrocarbon receptor (AhR), promoting nuclear translocation and deubiquitination in tumor‐reactive CD8 + T cells, thereby promoting CD8 + T cell exhaustion and HNSCC tumorigenesis (Lou et al. 2025). Commensal microorganisms have evolved with their host over time (Swain Ewald and Ewald 2020). Most protect their inhabitant from maladaptive changes by producing metabolites necessary for normal tissue function and competing with potential pathogens (Orlandi et al. 2019). Moreover, host immune system balance is influenced by environmental factors, including pH, chemical composition, and nutrient availability (Zhu et al. 2017). Oral normal bacterial flora includes phyla Firmicutes, *Proteobacteria, Bacteroidetes, Actinobacteria, Fusobacteria, Streptococcus, Haemophilus*, and *Prevotella* (Orlandi et al. 2019; Schmidt et al. 2014). Despite the usual presence of Fusobacteria in the oral cavity, when dysbiosis occurs, the expression of *fadA* from *Fusobacterium nucleatum (F. nucleatum)* acts as a virulence gene. FadA binds to E‐cadherin, which initiates intracellular signaling cascades, including the β‐catenin pathway, which can lead to upregulating oncogenes involved in cell proliferation, such as *cyclin D1* and *c‐Myc*, and the NF‐κB pathway, making epithelial cells more prone to becoming cancerous (Ye et al. 2024; Morrison 2024). Various microbiomes interact with oncogenic pathways as well; for instance, *P. gingivalis* initiates epithelial‐to‐mesenchymal transition (EMT) in gingival epithelial cells via Zinc finger E‐box‐binding homeobox 1 (ZEB1) (Sztukowska et al. 2016). These interactions illustrate how distinct oral microbiomes may contribute to tumor progression through specific epigenetic and transcriptional regulation rather than through direct genomic alterations. This can affect our therapeutic approach towards HNSCCs.

In addition, dysbiosis promotes the initiation and progression of HNSCC by sustaining chronic inflammation, modulating immune responses, and generating carcinogenic byproducts such as Acetaldehyde and Nitrosamines (Monteiro et al. 2024; Kwak et al. 2024).

Oral microorganisms are unique to individuals and are influenced by age, lifestyle, oral pH, and geographic location (Burcher et al. 2022). The mechanisms by which the microbiome changes over time remain incompletely understood; however, periodontal diseases are a significant factor in altering the oral microbiome (Garud and Pollard 2020).

Dental caries, though the age‐standardized prevalence rate has decreased over the years, 3.09 billion people still suffered from it in 2019, making it a leading cause of oral cavity dysbiosis (Qin et al. 2022). Periodontal disease is also significantly associated with at least one decayed tooth (Romandini et al. 2024). The dramatic shift in oral microbiota caused by periodontal disease‐related bacteria can be another pivotal factor. Furthermore, most key microbiomes affecting HNSCCs involve dental‐associated diseases, including *P. gingivalis* and *F. nucleatum*, *Treponema denticola*, *Prevotella*, and *Capnocytophaga* species (Sahin and Sonmezer 2024). Evidence shows that patients with a periodontitis diagnosis are 3.7‐fold more prone to oral SCC (OSCC) (Orlandi et al. 2019); furthermore, the OSCC of those with periodontitis is poorly differentiated compared to those without periodontitis (Shin et al. 2019). Periodontal pathogens were also more abundant in gingival SCC (GSCC) tissues, whereas saliva and soft tissue contained more bacteria associated with healthy periodontium (Li et al. 2020). In addition, a recent systematic review advocated that key periodontal pathogens, including *P. gingivalis* and *F. nucleatum*, contribute to OSCC progression through mechanisms involving inflammation, epithelial‐to‐mesenchymal transition (EMT), and immune evasion, providing robust evidence for the periodontitis‐oral cancer link (Pigossi et al. 2025).

Given the central role of oral dysbiosis in shaping oncogenic pathways, in this study, we delve into details of how periodontitis‐associated bacteria can alter the HNSCC′s profile in precise sections of the host immune system′s interaction with these bacteria and their association with HNSCC. Moreover, we review the interactions of the mentioned bacteria with oncogenic pathways and pathways linking bacteria to genetic mutations. We also explore epigenetic modifications induced by the mentioned bacteria and bacteria′s virulence genes. Finally, we provide a future perspective and a section on clinical implications to discuss how to engage with these data in clinical practice. This review is divided into eight sections. Following the introduction, Section Materials and Methods, 2 outlines our literature search strategy. Section Results, 3 provides a detailed analysis of specific periodontal pathogens and their virulence strategies. Section Conclusions, 4 combines these insights to demonstrate how bacterial activity affects key oncogenic pathways. Section 5 explores epigenetic changes caused by periodontal bacteria. Section 6 discusses current clinical applications and highlights key areas for future research. Section 7 emphasizes immediate clinical applications that can be incorporated into current practice. Lastly, Section 8 is the conclusion of this review.

## 2 Methods: Narrative Review and Literature Search Strategy

This article is a narrative review. A comprehensive search was conducted in PubMed and Google Scholar to identify studies linking HNSCC/OSCC with periodontitis‐associated bacteria and oral dysbiosis/microbiome. The last search was performed on January 28, 2026. The PubMed search used the following Boolean strategy (MeSH + free‐text terms):

1. HNSCC/OSCC block:
  “Squamous Cell Carcinoma of Head and Neck”[MeSH Terms] OR “head and neck squamous cell carcinoma”[All Fields] OR “hnscc”[All Fields] OR “head and neck squamous cell carcinomas”[All Fields] OR “hnsccs”[All Fields] OR “head and neck carcinogenesis”[All Fields] OR “head and neck carcinoma”[All Fields] OR “oral squamous cell carcinoma”[All Fields] OR “oral squamous cell carcinomas”[All Fields] OR “oscc”[All Fields] OR “osccs”[All Fields] OR “nasopharynx squamous cell carcinoma”[All Fields] OR “hypopharynx squamous cell carcinoma”[All Fields] OR “hypopharynx squamous cell carcinomas”[All Fields] OR “larynx squamous cell carcinoma”[All Fields] OR “oropharynx squamous cell carcinoma”[All Fields] OR “oropharynx squamous cell carcinomas”[All Fields] OR “lingual squamous cell carcinoma”[All Fields] OR “lingual squamous cell carcinomas”[All Fields] OR “oral cavity squamous cell carcinoma”[All Fields] OR “gingival squamous cell carcinoma”[All Fields] OR “gscc”[All Fields]
2. Bacteria/microbiome/periodontitis block:

“Porphyromonas gingivalis”[MeSH Terms] OR “Fusobacterium nucleatum”[MeSH Terms] OR “Capnocytophaga gingivalis”[Supplementary Concept] OR “Fusobacterium nucleatum”[All Fields] OR “f nucleatum”[All Fields] OR “Porphyromonas gingivalis”[All Fields] OR “p gingivalis”[All Fields] OR “oral microbiomes”[All Fields] OR “oral microbiome”[All Fields] OR “oral cavity dysbiosis”[All Fields] OR “oral dysbiosis”[All Fields] OR “dysbiosis”[All Fields] OR “treponema denticola”[All Fields] OR “prevotella”[All Fields] OR “capnocytophaga”[All Fields] OR “periodontal pathogens”[All Fields] OR “periodontal pathogens a actinomycetemcomitans”[All Fields] OR “periodontal pathogens actinobacillus”[All Fields] OR “periodontal pathogen”[All Fields] OR “Capnocytophaga gingivalis”[All Fields] OR “c gingivalis”[All Fields] OR “aggregatibacter”[All Fields] OR “eikenella corrodens”[All Fields] OR “filifactor”[All Fields] OR “treponema denticola”[All Fields] OR “f nucleatum polymorphum”[All Fields] OR “streptococcus gordonii”[All Fields] OR “periodontitis”[All Fields] OR “periodontitis microbiome”[All Fields] OR “periodontitis microbiota”[All Fields]

Reference lists were also screened to identify additional eligible studies.

Due to the narrative design of this review, we did not use formal systematic review methods, such as the PRISMA workflow or risk‐of‐bias scores, which could lead to selection bias and affect reproducibility. To compensate, we performed an extensive search, manually reviewed reference lists, and carefully assessed the strength of the evidence, making distinctions between association and causality when possible. However, it is important to interpret these results within the context of a narrative synthesis, acknowledging its inherent limitations. Table 1 summarizes the key distinctions between associative and causal evidence as applied throughout this review.

**Table 1:** Distinguishing association from causality in microbiome‐HNSCC research.

Association	Causality
Observational findings (e.g., bacterial enrichment in tumor tissues)	Demonstrated mechanistic relationships (e.g., virulence factors activating specific pathways)
Correlative data from human studies	Experimental evidence from in vitro and animal models
May be confounded by smoking, alcohol, HPV status, or oral hygiene	Establishes direct biological effects

While many studies highlight links between bacteria related to periodontitis and HNSCC, establishing causality needs: (1) reliable epidemiological data, (2) biological plausibility backed by mechanistic evidence, (3) evidence of dose‐response relationships, and (4) experimental validation. This review combines both associative and causal findings, indicating where mechanistic research supports causal claims and where confounding factors might still influence results.

## 3 Periodontitis‐Associated Bacterial Genomes and Virulence Factors

Periodontitis‐associated bacteria have unique mechanisms that aid HNSCC progression and share common pathways, including immune evasion and the promotion of a tumor‐promoting environment, mediated by lipopolysaccharide (LPS). Moreover, they can damage host DNA directly with their toxin (see Table 1).

Microorganisms are detected via germline‐encoded pattern‐recognition receptors (PRRs) and pathogen‐associated molecular patterns (PAMPs).

Most gram‐negative bacteria, including all the bacteria discussed in this article, have LPS as their endotoxin, which is located outside the cell wall (Wang and Quinn 2010). LPS contains three components of lipid A, a core oligosaccharide on which the attachment of LPS to the cell wall depends, and a polysaccharide part of the O‐antigen (Bertani and Ruiz 2018). The differences in the lengths of LPS fatty acid chains among various bacteria make each unique and determine how the host immune system responds to them (Steimle et al. 2016). Each LPS stimulates innate immune responses by attaching to its specific Toll‐like receptors (TLR) (Gronow and Brade 2001). Based on the strength of LPS in immunogenicity, inflammatory responses occur. Generally, lipid A binding to TLR4 induces innate immune responses in gram‐negative bacteria, contributing to the robust secretion of pro‐inflammatory cytokines, such as IL‐6, TNF‐α, and interferon (IFN) (Herath et al. 2013). This binding also regulates transcriptional factors such as activator protein 1 (AP‐1) and nuclear factor κB (NF‐κB), which are critical to HNSCC progression by influencing inflammation, cell survival, proliferation, angiogenesis, and immune evasion (Tan and Kagan 2014). However, this can differ in bacteria with atypical LPSs. For instance, regarding *P. gingivalis* LPS, although recognized by both TLR2 and TLR4, its lipid A binds to TLR2, thus provoking a weaker immune response, more like manipulating the host′s innate immunity (Bostanci and Belibasakis 2012; Hajishengallis et al. 2009). *P. gingivalis* LPS under hypoxia elevates NLRP3 and NF‐κB activity, increasing IL‐1β production and inflammation. Cytosolic complexes such as the NLRP3 inflammasome are key regulators of inflammatory responses. This is while in normal oxygen conditions, *P. gingivalis* LPS is negatively regulated by NLRP3, leading to controlled levels of IL‐1β (Kajiwara et al. 2017; Cheng et al. 2017).

Notably, various bacterial species in an environment are essential for significantly inducing cancer progression or inflammation (Sztukowska et al. 2016).

Intriguingly, the LPS of *P. gingivalis* can induce PD‐L1 expression and partial EMT in TLR4‐expressing OSCC cell lines (Omori et al. 2024). Engagement of TLR4 by *P. gingivalis* LPS recruits the adaptor protein MyD88. This activation of the IKK complex leads to phosphorylation and degradation of IκB, which normally sequesters NF‐κB in the cytoplasm. Consequently, the transcription factor NF‐κB, particularly the p65 subunit, translocates to the nucleus (Golusińska‐Kardach et al. 2025). The binding of NF‐κB to specific elements in the promoter region of the *CD274* gene, which encodes PD‐L1, increases its transcription and protein expression on the cancer cell surface. Upregulated PD‐L1 levels on tumor cells engage PD‐1 receptors on tumor‐infiltrating T cells and NK cells, delivering an inhibitory signal that reduces their cytotoxic activity, thereby promoting immune evasion. This process directly links chronic bacterial infections with resistance to the host immune system and could affect responses to ICI therapy (Sobhani et al. 2025; Strati et al. 2025).

Single‐cell RNA sequencing defined new roles for bacteria in the TME of the OSCCs. For instance, the heterogeneous distribution of *F. nucleatum* within the TME of OSCSS samples indicates that the TME can affect discrete cells. Moreover, intracellular bacteria can induce gene expression patterns in cancer cells that are associated with invasion, metastasis, alterations in DNA damage response mechanisms, and cell dormancy. In addition, invasive bacteria can attract myeloid cells into the TME. Thereby activating the JAK‐STAT signaling pathway, leading to T‐cell exclusion and secreting chemokines and interleukins, promoting a pro‐inflammatory environment (Galeano Niño et al. 2022).

Despite shared mechanisms among periodontitis‐associated bacteria for manipulating the immune system, each bacterium has distinct pathways that promote HNSCCs, which we briefly discuss in the subsections below.

### 3.1 Capnocytophaga gingivalis

*Capnocytophaga gingivalis*, a species of the *Capnocytophaga* genus, as a periodontal pathogen, is an anaerobic, gram‐negative bacterium associated with gingivitis and periodontitis, particularly in patients with compromised oral hygiene.

Recent data show that *C. gingivalis* invades OSCC tissues. According to Marger et al. (2005), *C. gingivalis* is among the three most prevalent species among 40 in the saliva of patients with OSCC. Other studies have shown the same result. Fluorescence in situ hybridization (FISH) also detected the high presence of *C. gingivalis* in the OSCC tissue (Zhu et al. 2024). In 160 samples of HNSCC tissues from various origins collected in TCRA, 51.23% contained the *Capnocytophaga* genus.

In addition to the presence of *C. gingivalis* in the HNSCC tissues, its particular virulence factors and bacterial proteins can potentially lead to HNSCC both directly and indirectly. Genome analysis of *C. gingivalis* identified over 5230 genes, with 913 encoding unique proteins. Key proteins, such as DNA gyrase subunit A and Lon protease 2, have been implicated in chronic inflammation and extracellular matrix (ECM) degradation (Salman et al. 2023). Lon protease 2 is an ATP‐dependent protease detected in several bacteria, including *C. gingivalis*. It plays a role in bacterial overall health and adaptation to hostile conditions through protein quality control (Kirthika et al. 2023). This protein also contributes to the degradation of host proteins, including ECM components, thereby facilitating tumor cell invasion and metastasis (Coleman et al. 2021). Moreover, several proteins play a vital role in fatty acid synthesis and are therefore involved in LPS synthesis, such as acetyl‐Coenzyme A carboxylase carboxyl transferase subunit alpha.

In a study by Zhu et al. (2024), the impact of *C. gingivalis* on the OSCC cell lines stimulated by *C. gingivalis* was investigated. These cell lines exhibited fibroblastoid phenotypes with decreased E‐cadherin and increased vimentin/SNAIL levels, consistent with EMT induction.

*Capnocytophaga gingivalis* also produces tissue‐destroying hydrolytic enzymes, contributing to the destruction of the gingival soft tissue and alveolar bone. The aminopeptidase can be responsible for collagen degradation and bradykinin formation (Spratt et al. 1995). This is essential for the progression of periodontitis; however, in the case of HNSCC, it can also facilitate invasion (Sukmana et al. 2024).

While evidence supports the role of *C. gingivalis* in HNSCC progression, the underlying molecular mechanisms remain incompletely understood. Further investigations are needed to understand these mechanisms, which may reveal novel biomarkers and therapeutic targets in HNSCC management.

### 3.2 Fusobacterium nucleatum

*Fusobacterium nucleatum* is a gram‐negative obligate anaerobic bacterium existing parasitically in both normal and infected oral flora. It plays various roles in diseases ranging from mild to severe gingivitis and periodontitis to angina, lung abscess, chronic otitis, and gastrointestinal diseases. Its strong pathogenicity led to periodontitis and bone loss in mice infected with *F. nucleatum alone*, even though periodontitis requires multiple bacterial species for its development (Chaushu et al. 2012). Notably, *F. nucleatum* is detected more often in the saliva of patients with periodontitis than in healthy individuals (Zhou et al. 2015). It can also facilitate interactions between primary and secondary colonizer bacteria, thereby increasing the risk of cancerogenesis, via adhesive components such as Aid1, CmpA, Fap2, FomA, FadA, and RadD (Rizzato et al. 2019; Han 2015). The most studied and essential virulence factor of *F. nucleatum* is FadA, encoded by *fadA*, which is the only adhesive component of *F. nucleatum* that aids in its binding to host cells and plays a significant role in HNSCC tumorigenesis. FadA binding to the E‐cadherin leads to activating the β‐catenin signaling pathway. β‐catenin translocation to the nucleus activates the p38MAPK pathway, thus enhancing the secretion of matrix metalloproteinase‐9 (MMP‐9) and MMP‐13. This induces ECM degradation and facilitates metastasis (Chattopadhyay et al. 2019). Rubinstein et al. (2013) showed that preventing FadA binding to E‐cadherin can reduce the tumorigenic responses of colorectal cancer. Interestingly, the FadA expression level is reduced when the outer membrane vesicles (OMVs) of *P. gingivalis* interfere with oral epithelial cells (Zhang et al. 2022). This is while *F. nucleatum* improves the adhesion of *P. gingivalis* to human gingival epithelial cells (Li et al. 2015).

*Fusobacterium nucleatum*, contrary to *P. gingivalis*, which is significantly involved in various stages of HNSCCs, mostly plays its part by upregulating different pro‐inflammatory cytokines, including TNF‐α, IL‐6, IL‐8, IL‐10, and IL‐12 (Han 2015).

In addition to inducing pro‐inflammatory cytokines, *F. nucleatum* employs virulence factors such as Fap2 to modulate the immune response, thereby promoting tumor formation. Fap2 enables *F. nucleatum* to inhibit NK cells and tumor‐infiltrating lymphocytes via direct binding, contributing to immune evasion (Hussan et al. 2017; Zhang et al. 2018).

Moreover, *F. nucleatum* can influence the TME through vesicle‐mediated signaling. Li et al. (2025) reported that *F. nucleatum‐*derived OMVs alter tryptophan metabolism in tumor‐associated macrophages (TAMs), thereby enhancing resistance to immune checkpoint therapies. As a result, macrophages exhibit high AHR+ immunosuppressive activity. Furthermore, expression of immune checkpoint molecules, including TIM3/HAVCR2, CD47, and PD‐L2/PDCD1LG2, increases, and infiltration of CD8 + T‐cells decreases. An OMV‐related macrophage signature demonstrated predictive power for immunotherapy response in both HNSCC and colorectal cancer datasets, with an AUC of approximately 0.75–0.76, highlighting OMV‐driven microbial effects as an important factor influencing variability in treatment response.

Beyond its direct effects on host‐cell adhesion and immune evasion, *F. nucleatum* regulates microRNA (miRNA) expression, complicating its role in cancer progression. *F. nucleatum* modulates miRNA expression, influencing cancer progression through various molecular pathways. However, the exact mechanisms are yet to be fully elucidated. It is proven that in laryngeal SCC, *F. nucleatum* can upregulate the miR‐205‐5p expression by activating innate immune signaling pathways. The miR‐205‐5p suppresses the expression of alcohol dehydrogenase 1B (ADH1B) and transforming growth factor β receptor 2, leading to ethanol metabolism reprogramming and promoting EMT in laryngeal SCC (Hsueh, Huang et al. 2022). In another study, the researchers showed that *F. nucleatum* increased miR‐205‐5p expression, which suppressed MLH1, MSH2, and MSH6, via TLR4‐ and MYD88‐dependent innate immune signaling pathways in HNSCC tissues. This inhibited the DNA mismatch repair process, thus inducing more genomic instability in HNSCC cells (Hsueh, Lau et al. 2022).

*Fusobacterium nucleatum* genomic adaptability, virulence factors, and modulation of host immune and signaling pathways highlight its role in HNSCC progression. HNSCCs with a high prevalence of *F. nucleatum* were more likely to be detected in older individuals, with less relation to the use of alcohol and tobacco (Neuzillet et al. 2021). This, combined with the aforementioned roles of *F. nucleatum* in HNSCC tissues, makes *F. nucleatum* a strong candidate as a biomarker and therapeutic target in cancer management.

### 3.3 Porphyromonas gingivalis

*Porphyromonas gingivalis* is a gram‐negative, obligatory anaerobic, non‐motile, rod‐shaped bacterium often known as a keystone pathogen in periodontitis. It disrupts the microbial balance in the oral cavity, driving inflammation and tissue destruction. Research has linked *P. gingivalis* with several systemic conditions, including cardiovascular diseases, rheumatoid arthritis, Alzheimer′s disease, and HNSCCs.

The genome of *P. gingivalis* exhibits high genetic variability, enabling it to adapt to diverse microenvironments and interact with host cells. The main virulence factors of *P. gingivalis* are fimbriae, gingipains, LPS, nucleoside diphosphate kinase (NDK), and its capsule, encoded by specific gene clusters. These factors enable the bacterium to modulate host immune responses and enhance survival.

For instance, *P. gingivalis fimA* encoding fimA (one type of fimbriae on its cell) facilitates adhesion and invasion of epithelial cells by upregulating zinc finger E‐box‐binding homeobox‐1 (Zeb‐1) via PI3K/Akt and β‐catenin signaling pathways. This leads to repression of E‐cadherin production, enhanced vimentin expression, and upregulation of MMP1, MMP7, and MMP9 (Sztukowska et al. 2016). The *rpgA, rpgB*, and *kgp* genes encode gingipains, which further induce EMT by activating Zeb2 via β‐catenin‐ and FOXO1‐dependent pathways (Ohshima et al. 2019). ProMMP‐9 expression can be affected by *P. gingivalis* by activating the ERK1/2‐Ets1, p38/HSP27, and PAR2/NF‐KB pathways. Subsequently, its Gingipains directly cleave ProMMP‐9, producing activated MMP‐9, which facilitates EMT and its penetration into the lymphatic system (Inaba et al. 2014).

LPS, a hallmark of *P. gingivalis* virulence, is highly heterogeneous, each leading to a particular immune response (Trent et al. 2006). For instance, genes in the *waa* locus encode isoforms of *P. gingivalis* LPS that disrupt PRRs and aid the immune evasion process (Ding et al. 2017). Moreover, *LpxF* is responsible for modifying the lipid A component of LPS, thus altering the bacterium′s interaction with the host immune system (Alaei et al. 2024). A‐LPS regulates the production of IL‐1α, IL‐1β, IL‐6, and IL‐8. In addition, penta‐acylated *P. gingivalis* LPS regulates MMP‐3 expression in HGFs, destroying the ECM (Herath et al. 2013).

While *P. gingivalis* aids HNSCC progression by fostering an inflammatory environment, it can also prevent it. It is proven that specific isoforms of *P. gingivalis* LPS can upregulate human beta‐defensins (hBD‐1, hBD‐2, hBD‐3) in human epithelial cells (Lu et al. 2009). The hBD‐1, which provides baseline antimicrobial protection in the oral cavity, is downregulated in various cancers, including HNSCCs (Han et al. 2014). However, chronic inflammation driven by hBD‐2 can promote carcinogenesis by facilitating angiogenesis and recruiting immune cells that create a pro‐tumorigenic inflammatory milieu (Kompuinen et al. 2023).

*Porphyromonas gingivalis* hijacks host cellular processes to suppress apoptosis, guaranteeing survival within the host cell by activating the JAK/STAT and PI3K/Akt pathways (Yao et al. 2010). Moreover, NDK prevents P2X7‐mediated apoptosis and phosphorylates heat shock protein 27. This contributes to the inactivation of Bax, a pro‐apoptotic protein, thus preventing mitochondrial apoptotic pathways (Yilmaz et al. 2008). This genomic adaptability ensures prolonged intracellular persistence (Table 2).

**Table 2:** Periodontitis‐associated bacteria and their potential role in HNSCC progression.

Genus/Species	Role in HNSCC	Ref. no.
*Aggregatibacter actinomycetemcomitans*	Detected among species profiled in OSCC salivary microbiota studies; periodontopathogen with pro‐inflammatory potential.	Mager et al. (2005)
*Capnocytophaga gingivalis*	Enriched in OSCC saliva (candidate diagnostic marker). Shown to promote oral cancer phenotypes (EMT markers; ↓E‐cadherin, ↑vimentin/SNAIL).	Mager et al. (2005) and Zhu et al. (2024)
*Eikenella corrodens*	Included among species screened in OSCC salivary microbiota profiling studies (candidate OSCC‐associated taxa).	Mager et al. (2005)
*Fusobacterium nucleatum*	FadA binds E‐cadherin → β‐catenin/Wnt signaling; ↑MMPs, proliferation and invasion. Induces DNA damage/DDR changes (e.g., Ku70/p53) and EMT programs; can activate pro‐tumor pathways (e.g., YAP). Immune evasion via Fap2–TIGIT interactions; inflammatory cytokine induction.	Rubinstein et al. (2013), Kwak et al. (2024), Uitto et al. (2005), Geng et al. (2020), Martin (2020), and Yamamoto et al. (2023)
*Porphyromonas gingivalis*	LPS/TLR signaling induces PD‐L1 and invasive/partial EMT phenotypes in OSCC. Promotes invasion via pro‐MMP‐9 induction/activation; dysregulates oncogenic miRNAs. Activates PI3K/Akt and EMT programs (e.g., ZEB1); promotes oral carcinogenesis in vivo. Immune modulation: upregulates B7‐H1/B7‐DC receptors.	Omori et al. (2024), Inaba et al. (2014), Yao et al. (2010), Groeger et al. (2011), Kwak et al. (2024), Sztukowska et al. (2016), Chang et al. (2019), Zang et al. (2025), and Wu et al. (2018)
*Prevotella intermedia*	Orange‐complex member associated with increased HNSCC risk. Promotes OSCC progression (e.g., via ISG15 upregulation; tumor‐suppressor inhibition).	Kwak et al. (2024), Qin et al. (2024), and Zhou et al. (2024)
*Tannerella forsythia*	Red‐complex member associated with increased HNSCC risk. Detected among taxa profiled in OSCC salivary microbiota studies.	Mager et al. (2005) and Kwak et al. (2024)
*Treponema denticola*	Red‐complex member associated with increased HNSCC risk. Promotes OSCC development via TGF‐β signaling.	Kwak et al. (2024) and Peng et al. (2022)

Despite these mechanisms, several genes help *P. gingivalis* survive in the oral cavity, promoting chronic inflammation. To name a few, genes that encode proteins mediating bacterial adhesion to host tissues, including *Hemagglutinin* genes (*HagA, HagB*, and *HagC*) and *Sod*, which neutralizes reactive oxygen species (ROS) (Carvalho‐Filho et al. 2016; Lynch and Kuramitsu 1999). Furthermore, *OmpA* and *OmpH*, which encode outer‐membrane proteins, contribute to adhesion, invasion, and immune modulation (Naylor 2016).

The genomic landscape of *P. gingivalis* supports its virulence, qualifying it to manipulate host pathways and contribute to HNSCC progression through multiple mechanisms. However, the molecular mechanisms behind many of *P. gingivalis′s* effects on HNSCC progression are unclear. For instance, *P. gingivalis* activates B7 homolog 1 in OSCC cells, causing T‐cell apoptosis, but the mechanism remains to be elucidated (Groeger et al. 2011).

**Figure 1:**
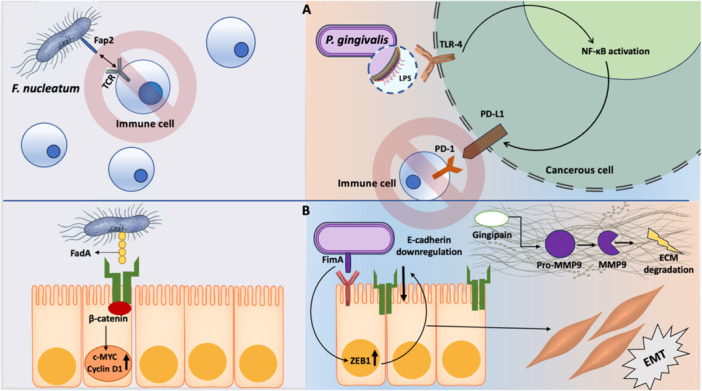
Virulence mechanisms of key pathogens.

Most periodontal bacteria are gram‐negative obligate/facultative anaerobes. Due to hypoxia, the TME alters the diversity of these bacteria. Bacteria involved in periodontitis pathogenesis induce chronic inflammation and immune dysregulation and manipulate signaling pathways, including β‐catenin and NF‐κB, promoting EMT, genomic instability, and a pro‐tumorigenic microenvironment. Their virulence factors, such as adhesins, proteases, and unique LPS, determine how bacterial genomes evade host immunity and induce oncogenic changes. Other periodontal bacteria may also affect HNSCCs via similar pathways. This understanding underscores the importance of oral health in cancer prevention and the potential of these bacteria as biomarkers and therapeutic targets in the management of HNSCC. Future research will be essential to elucidate the precise molecular mechanisms and to explore interventions that translate this knowledge into improved clinical outcomes (see Figure 1).

## 4 Periodontitis‐Associated Bacteria and Oncogenic Pathways of HNSCC

The progression of HNSCC is linked to bacteria associated with periodontitis. These microorganisms facilitate oncogenesis by interacting with host pathways that align with the hallmarks of cancer, including sustaining proliferative signaling, evading growth suppressors, promoting angiogenesis, and modifying the TME. This section explores the mechanisms by which key periodontitis‐associated bacteria—specifically *F. nucleatum, P. gingivalis*, and *Prevotella intermedia (P. intermedia)*—enhance HNSCC progression. By linking their effects to established oncogenic pathways, this section highlights the potential of these bacteria as therapeutic targets and biomarkers for diagnosis.

Among *F. nucleatum* subspecies, *F. nucleatum polymorphum* is the most enriched in OSCC tissues, underscoring the role of bacterial subspecies in driving oncogenic proliferation. Yamamoto et al. (2023) demonstrated that treating tongue cancer mouse models with *F. nucleatum polymorphum* isolated from patients with oral cancers stimulates the oncogenic proliferation of oral epithelial cells by increasing the number of Ki‐67‐positive cells and also expanding the nuclear Yes‐Associated Protein (YAP) localization area within the mouse tongue epithelium. These findings suggest that the bacterium drives the early steps of oral cancer progression by activating cell proliferation and survival pathways.

Similarly, *P. gingivalis* contributes to OSCC proliferation through mechanisms involving cell cycle regulation and modulation of miRNA.

According to Chang et al. (2019), *P. gingivalis* may promote OSCC proliferation by modulating cyclin D1 expression. In the Tca8113 OSCC cell lines infected with *P. gingivalis*, AP‐1 elevation led to the increase of cyclin D1, a key regulator of the G1‐to‐S phase transition. miR‐21 was also found to be upregulated in *P. gingivalis*‐infected OSCC cells. miR‐21 targets programmed cell death 4 (PDCD4), a tumor suppressor that inhibits cell proliferation and promotes apoptosis. Thus, miR‐21 upregulation downregulates PDCD4, which typically restrains AP‐1 activity, thereby forming a miR‐21/PDCD4/AP‐1 negative‐feedback signaling pathway that contributes to cell‐cycle progression and proliferation.

Beyond its short‐term effects on cell cycle progression, prolonged *P. gingivalis* infection induces expression of cancer stem cell markers, thereby further enhancing tumor proliferative capacity. Notably, long‐term infection by *P. gingivalis* elevates the expression of CD44 and CD133, both stem cell markers (Ha et al. 2015). A recent study also revealed that *P. gingivalis* can enhance the stem‐like features of OSCC by influencing stearoyl‐CoA desaturase‐1 (SCD1)‐ dependent lipid synthesis through an NOD1/KLF5 pathway. Both genetically and pharmacologically, inhibition of SCD1 reduces cancer stemness and chemoresistance in vitro and slows tumor growth in vivo, highlighting the connection between periodontal infection, metabolic changes, and the support of cancer stem cells (CSCs) (Zang et al. 2025).

Interestingly, the influence of *F. nucleatum* on the cell cycle appears to vary depending on the host′s genetic context, underscoring the complexity of these interactions.

In a study by Zhang et al. (2020), *F. nucleatum* infection in the SCC‐9 cell line did not affect cell proliferation, as assessed by flow cytometry. While both the SCC‐9 and the Tca8113 are derived from tongue SCCs, the different results may be due to their distinct genetic background. Tca8113 is well characterized for its high proliferation rate and invasive properties, potentially making it more responsive to cell‐cycle alterations. Moreover, the Tca8113 cell line, unlike the SCC‐9 cell line, harbors a functional p53, whereas in SCC‐9, p53 is mutated and nonfunctional. Notably, the latest study analyzed the cell cycle using flow cytometry, which provides a snapshot of cell‐cycle distribution, but it may not detect subtle molecular changes.

*Treponema denticola* (*T. denticola*) has also been proven to invade the Cal‐27 cell line and enhance cell proliferation. However, the precise mechanism remains to be elucidated (Peng et al. 2022).

These findings illustrate the multifaceted roles of periodontitis‐associated bacteria in promoting OSCC proliferation, highlighting shared and distinct mechanisms that may vary with host and tumor context.

While sustaining proliferative signaling underpins tumor growth, destabilizing genomic integrity and suppressing growth inhibitors by periodontitis‐associated bacteria accelerate oncogenic processes in HNSCC.

Yao et al. (2021) explored this interplay, demonstrating how periodontal pathogens, including *P. gingivalis* and *F. nucleatum*, promote OSCC by manipulating the ATR‐CHK1 DNA damage response pathway. According to their investigation, mice with *in situ* OSCC, stimulated with *P. gingivalis* and *F. nucleatum*, showed prolonged S‐phase and shortened G1‐phase compared with the control group. Moreover, compared to the control group, upregulated expression levels of γ‐H2AX, p‐ATR, RPA32, CHK1, and RAD51 were detected, while the phosphorylation level of CHK1 (p‐CHK1) was downregulated, leading to more genomic instability. Moreover, *F. nucleatum* infection of the Tca8113 (tongue SCC cell line) resulted in accelerated cell cycle progression and downregulation of p27. Moreover, it leads to downregulation of Ku70 and wild‐type p53, thereby promoting the proliferation of Tca8113 by inducing DNA double‐strand breaks (Geng et al. 2020). The p53 downregulation caused by *F. nucleatum* can also be through activation of the Wnt/NFAT pathway, particularly upregulation of *Wnt5a* and NFATc3 (Da et al. 2021).

*Porphyromonas gingivalis* also promotes OSCC growth by inhibiting apoptosis and promoting autophagy. Autophagy is a double‐edged sword in cancer, as it can help established tumors survive stressful conditions (Yuan et al. 2024).

Controversially, *P. gingivalis*‐derived phosphor‐ethanolamine dihydroceramide (PEDHC) can affect OSCC cell behavior by downregulating *acid ceramidase (ASAH1)* mRNA expression. ASAH1 encodes acid ceramidase, an enzyme that breaks down ceramides into sphingosine and free fatty acids (Doan et al. 2017). Ceramides are bioactive lipids with antiproliferative, pro‐apoptotic, and anti‐migratory effects. Elevated ceramide levels can suppress tumor growth and progression. Although OSCC cells may tolerate low ASAH1 expression and ceramide accumulation to some extent, this creates metabolic vulnerability. Thus, *P. gingivalis*‐derived PEDHC accumulates ceramides, thereby suppressing cell proliferation and migration. Moreover, PEDHC downregulated gene expression in the basement membrane and ECM degradation, including MMP‐2, ADAM‐17, and IL‐6 (Yamada et al. 2023). Notably, PEDHC synthesis is susceptible to bacterial strains. A *P. gingivalis* mutant strain unable to synthesize PEDHC (ΔPG1780) fosters markedly more aggressive OSCC cell proliferation and migration than the wild‐type W83 strain (Yamada et al. 2023). However, the pro‐carcinogenic characteristics of *P. gingivalis* are driven by the combined activity of its other virulence factors, facilitating tumor progression through chronic inflammation, immune evasion, induction of EMT, and inhibition of apoptosis (Starska‐Kowarska 2025). The pro‐carcinogenic effects of *P. gingivalis* in vivo outweigh the PEDHC‐mediated pathway, which is one node in a complex interaction network. This underscores the importance of comprehensively evaluating bacterial pathogenesis, considering specific bacterial products, host cell type, and the broader TME. Moreover, from another perspective, since PEDHC induces ceramide accumulation in OSCC cells and ceramide is a key regulator of exosome biogenesis, it is plausible to hypothesize that *P. gingivalis* infection may also modulate exosome‐mediated communication in the OSCC TME (Yamada et al. 2023; Lu et al. 2021). Future studies are needed to directly measure exosome output from OSCC cells following exposure to PEDHC or *P. gingivalis* to test this hypothesis.

The multifaceted strategies used by *P. gingivalis* and *F. nucleatum* highlight their roles in promoting genomic instability, altering tumor metabolism, and creating a microenvironment that supports HNSCC progression.

Beyond impacting cell cycle regulation and genomic stability, periodontitis‐associated bacteria reshape cellular metabolism to meet the energy demands of proliferating cancer cells.

Sun et al. (2023) also showed that *F. nucleatum* is abundant in the invasive margins of OSCC tissues, highlighting its role in tumor invasion. Additionally, the group revealed that *F. nucleatum* impacts TAM formation by activating the GalNAc‐Autophagy‐TBC1D5 signaling pathway. This activation leads to GLUT1 aggregation, which enhances glycolysis, thus producing excess lactate and secretion outside the cell. High lactate levels in the TME promote TAM formation, thereby creating a microenvironment that is favorable for cancer progression.

*Fusobacterium nucleatum* influences the metabolism of HNSCC cells, contributing to tumor progression. This was investigated in a study coculturing AMC‐HN‐8 cells with *F. nucleatum*. The results displayed significant changes in the metabolism of AMC‐HN‐8 cells over time when cocultured with *F. nucleatum*. The purine metabolic pathway was the most altered, resulting in downregulation of purine degradation, thereby supporting cancer cell growth and survival. Another intriguing result was that adding uric acid, a byproduct of the purine degradation pathway, can reverse the tumor‐promoting effects of *F. nucleatum* and decrease intracellular ROS levels, as evidenced by the negative correlation between uric acid levels and *F. nucleatum* abundance in 113 HNSCC patients (Li et al. 2023).

*Porphyromonas gingivalis* promotes OSCC development by manipulating fatty acid metabolism and increasing free fatty acids in cancerous tissue and the serum. It also elevates the expression of lipid‐synthesizing enzymes, fatty acid synthase (FASN), and acetyl‐CoA carboxylase 1 (ACC1). The overexpression of these enzymes is associated with cancer progression, as cancer cells rely on increased lipid synthesis for energy and membrane production (Wu et al. 2018).

In addition to metabolic reprogramming, bacterial infections stimulate angiogenesis, a critical process that supports tumor survival and growth by ensuring nutrient and oxygen supply.

Several studies have shown that periodontitis‐associated bacteria can also promote angiogenesis in HNSCCs. *F. nucleatum* infection in H376 cell lines (derived from a stage 3 OSCC) led to upregulating the pro‐angiogenic chemokines MCP‐1 and VEGF‐A, contributing to capillary‐like tube formation in HUVEC cells (Selvaraj et al. 2024).

Similarly, *P. intermedia* plays a pivotal role in promoting angiogenesis through distinct molecular mechanisms. Intra‐tumoral injection of *P. intermedia* into a murine xenograft model significantly increased expression of *Interferon‐stimulated gene 15* (*ISG15*). ISG15 expression is primarily driven by type I interferon (IFN‐α/β) signaling. Chronic inflammation in the TME or viral mimicry pathways leads to sustained IFN production, which subsequently activates the JAK/STAT pathway. Phosphorylated STAT1/STAT2 complexes translocate to the nucleus and bind to interferon‐stimulated response elements (ISREs) in the ISG15 promoter, leading to its transcription (Chen et al. 2025). Higher *ISG15* expression is associated with increased Ki67 expression, thereby accelerating tumor cell proliferation. It is also associated with higher microvessel density, elevated cytokine levels, and the presence of pro‐tumor immune cells, such as M2 macrophages and Tregs (Qin et al. 2024). Additionally, sustained exposure to secreted ISG15 promotes CD8 + T‐cell dysfunction (exhaustion) in the OSCC TME, despite their recruitment. Notably, this dysfunction remains responsive to PD‐1 blockade, suggesting that ISG15 could serve as a biomarker of ICI benefit (Wu, Shao et al. 2025). The same result was achieved by using heat‐killed *P. intermedia* (Zhou et al. 2024).

With a well‐established nutrient and oxygen supply, bacteria further contribute to cancer progression by promoting invasion and metastasis through EMT and enhanced migration pathways.

As detailed in Section 3.1, individual periodontal pathogens independently induce EMT through distinct molecular mechanisms. Beyond these individual effects, the dual‐species community of *P. gingivalis* with *F. nucleatum* and *Streptococcus gordonii* (*S. gordonii*) further enhanced the ZEB1 expression, indicating a possible synergistic effect of multiple oral bacteria in driving EMT‐related pathways (Sztukowska et al. 2016). *S. gordonii* is a member of normal oral flora and can be known as a commensal bacterium; however, it acts as an early colonizer in periodontal diseases (Park et al. 2020). Notably, *P. gingivalis* strains lacking FimA showed a reduced ability to induce ZEB1, indicating that FimA‐mediated signaling is a key driver of this effect (Sztukowska et al. 2016).

Moreover, *P. gingivalis*, along with *F. nucleatum* and *T. denticola*, facilitates OSCC migration by activating Integrin alpha V and focal adhesion kinase (FAK). *T. denticola* specifically upregulates TLR‐2, TLR‐4, and MyD88. Interestingly, MyD88 expression was required for *T. denticola*‐mediated FAK activation and OSCC cell migration (Kamarajan et al. 2020).

This collective bacterial activity exemplifies how diverse pathogens converge on common pathways to promote metastatic potential in HNSCC cells.

Finally, these bacteria′s ability to manipulate the TME and modulate immune responses ensures a supportive niche for tumor survival and dissemination.

As mentioned, the inflammasome/IL‐1β pathway is involved in HNSCC progression and is also associated with *P. gingivalis* and *F. nucleatum*. In the H400 cell line exposed to *F. nucleatum* and *P. gingivalis* individually and in combination, NLRP3 expression was significantly upregulated, leading to enhanced expression of IL‐1β. In *P. gingivalis* and *F. nucleatum* individual samples, the upregulated IL‐1β resulted from AIM2 upregulation and POP1 downregulation. In combined samples of both *F. nucleatum* and *P. gingivalis*, POP1 downregulation did not occur, highlighting their opposing roles in the HNSCC progression (Aral et al. 2020).

*Fusobacterium nucleatum* can enhance the ability of OSCC cells to recruit macrophages and induce their M2 phenotype by elevating CXCL2 production by OSCCs. This chemokine is crucial for macrophage recruitment and for polarization to the M2 phenotype. CXCL2 suppression in OSCCs abrogates *F. nucleatum′s* ability to promote OSCC cell behaviors, including proliferation, migration, and macrophage recruitment (Nie et al. 2024).

**Figure 2:**
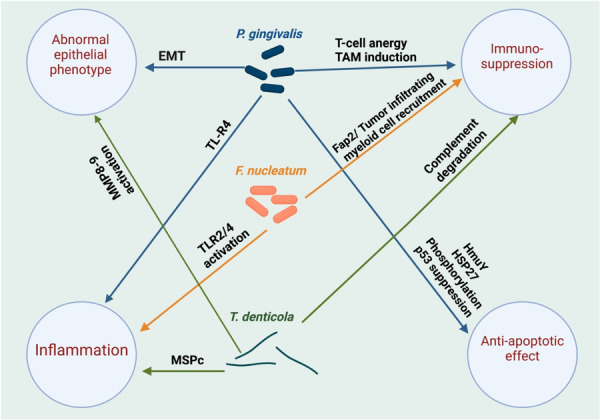
Periodontitis‐associated bacteria and oncogenic pathways of HNSCC.

Bacteria associated with periodontitis, such as *F. nucleatum, P. gingivalis, and P. intermedia*, play diverse and essential roles in the progression of HNSCC through their interactions with critical oncogenic pathways. These bacteria contribute to cancer characteristics, including persistent cell growth, resistance to growth inhibitors, and metabolic alterations, while promoting angiogenesis and increasing invasion and metastasis. Furthermore, they alter the TME by affecting immune responses and encouraging chronic inflammation. A deeper understanding of these interactions illuminates the molecular mechanisms driving HNSCC progression and creates opportunities for new therapeutic strategies and biomarker identification (see Figure 2).

### 4.1 Microbial Interactions in the Tumor Microenvironment: Synergy and Competition

As part of a complex community, microbes in the TME can interact, thereby intensifying or attenuating each other′s effects and influencing HNSCC progression.

*Fusobacterium nucleatum* can enhance the oral epithelium′s adhesion and invasion of *P. gingivalis*. *P. gingivalis* employs its fimbriae to attach to epithelial cells, a process that can be reinforced by FadA and FomA from *F. nucleatum*, thereby creating a more stable environment for colonization and consequently increasing inflammation and immune evasion (Yao et al. 2021).

They can enhance each other′s survival by sharing nutrients. For instance, *P. gingivalis* and *P. intermedia* can upregulate fatty acid synthase, resulting in increased free fatty acid production that *F. nucleatum* or *T. denticola* can utilize. This aids anaerobic bacteria in surviving in a hypoxic TME (Cai et al. 2024). Metabolic vulnerabilities in HNSCC cells influenced by bacteria could be targeted to slow tumor progression. Targeting metabolic pathways may prove effective as a new treatment strategy.

Furthermore, bacteria can form an immunosuppressive niche through their collaboration. For instance, *F. nucleatum* recruits M2 macrophages by upregulating CXCL2, while *P. gingivalis* evades immune surveillance by increasing PD‐L1 expression and avoiding immune checkpoints (Yao et al. 2021; Li et al. 2022). Modulating the oral microbiome may help reduce resistance to checkpoint inhibitor therapy in HNSCCs (Kamarajan et al. 2020).

On the other hand, they can also compete for TME resources and, as a result, alter the tumor progression. *P. gingivalis* downregulates FadA expression in *F. nucleatum* to limit its invading ability in epithelial cells. This may slow tumor progression (Wang et al. 2024). Since each individual has a unique oral microbiome, dynamic competition may contribute to distinct tumor behavior (Li et al. 2022).

Moreover, bacteria can alter microbial dominance within the tumor by eliminating other species. For instance, *P. gingivalis* gingipains can eliminate competitors, whereas *Streptococcus gordonii* produces bacteriocins that inhibit *F. nucleatum* and *P. gingivali*s, underscoring the significance of oral probiotics in modulating bacterial competition (Cai et al. 2024; Kamarajan et al. 2020).

## 5 Periodontitis‐Associated Bacteria and Epigenetic Aberrations in HNSCCS

Chronic periodontitis potentially alters gene expression epigenetically. Laser capture microdissection detected extensive epigenetic alterations in several chemokine and cytokine genes in epithelial cells stimulated by periodontal bacteria (Barros and Offenbacher 2014). Epigenetic alterations affect cancer development by modulating gene expression without altering the underlying DNA sequence.

### 5.1 DNA Methylation

DNA methylation is one of the main epigenetic mechanisms. It involves the methylation of 5mC within DNA, primarily in CpG regions, leading to gene silencing. DNA methyltransferase (DNMT) enzymes are primarily responsible for this process (Larsson et al. 2025). It has been shown that generalized periodontitis can lead to hypomethylation of *STAT5*, which plays an initiating role in the production of various cytokines (Azevedo et al. 2020). Moreover, altered DNA methylation levels in immune response‐related genes, including IL‐17C and chemokine ligand‐25, have also been detected in subjects with periodontitis (Schulz et al. 2016).

Gingival epithelial cells stimulated by *P. gingivalis* showed hypermethylation in the TLR2 promoter CpG island, leading to its downregulation and weakening of innate immunity (Benakanakere et al. 2015). The same epigenetic alteration in the *PTGS2* promoter region was also observed in patients with chronic periodontitis. PTGS2, also known as COX‐2, is an enzyme that plays a vital role in the biosynthesis of prostanoids, including prostaglandins (Zhang et al. 2010). Reduced PGE2 can potentially enhance the IL‐6/STAT3 pathway, thereby promoting the survival of pre‐malignant cells. According to Zhang et al. (2013), subjects with severe chronic periodontitis also demonstrated hypermethylation at two CpG sites within the TNFA promoter, one of which is significantly associated with reduced TNF‐α mRNA levels.

There have also been reports of hypomethylation in the promoter regions of pro‐inflammatory cytokines. Oliveira et al. (2009). reported that individuals with chronic periodontitis, regardless of their smoking status, exhibited higher levels of hypomethylation at the promoter site of IL‐8, resulting in its upregulation in gingival cells. *T. denticola* also leads to hypomethylation of the *MMP‐2* promoter in periodontal ligament stem cells; however, a few hypomethylations in this region may not have biological effects on *MMP‐2* gene expression (Miao et al. 2014).

### 5.2 Histone Modifications

The post‐translational histone modifications, including acetylation/deacetylation or methylation, can significantly impact gene expression by altering the chromatin structure.

Periodontal‐associated bacteria LPS, such as *P. gingivalis*, have been proven to downregulate the expression of DNMT1, DNMT3a, and JMJD3 in primary gingival fibroblasts and keratinocytes. Reducing these enzymes, which regulate epigenetic modifications, suggests that *P. gingivalis* alters tissue homeostasis by indirectly affecting epigenetic modifications (de Camargo Pereira et al. 2013).

*Porphyromonas gingivalis* infection also has direct effects. Histone acetylation was reduced in bone marrow macrophages of diet‐induced obese mice with *P. gingivalis* infection. This reduction suppresses the recruitment of NF‐kB transcription factors to the *TNF, IL‐10*, and *TLR2* promoter regions. This could further emphasize *P. gingivalis′s* multifaceted impact on the immune system (Zhou et al. 2011). Moreover, gingival epithelial cells stimulated with *P. gingivalis* and *F. nucleatum* showed altered *HDAC1, HDAC2*, and *DNMT1* expression. H3 Lys 4 methylation was also affected, which regulates the expression of Human β‐defensin‐2 and CC chemokine ligand 20 (Yin and Chung 2011).

Chronic inflammation increases ROS and pro‐inflammatory cytokines such as IL‐6, suppressing DNMTs and promoting hypomethylation, indicating an inflammation‐epigenetics feedback loop (Benakanakere et al. 2019).

The research indicates that bacteria silence immune‐recognition and anti‐inflammatory genes while activating pro‐inflammatory genes by hypermethylation and silencing key immune‐surveillance genes, such as TLR2 and TNFA, and by driving hypomethylation and activation of potent pro‐inflammatory mediators, including IL8 and MMP2 (Oliveira et al. 2009; Miao et al. 2014; Jurdziński et al. 2020). Notably, the direction of methylation (hyper‐ vs. hypo‐) is a result of the interplay of at least two major mechanisms: (a) Bacteria or their components can directly/indirectly recruit the epigenetic machineries. For instance, by downregulating DNMT expression, which would globally predispose the genome to hypomethylation (directly), or by recruiting specific DNMTs or histone modifiers to specific gene promoters (indirectly), leading to targeted hypermethylation and silencing (Lavu et al. 2015). (b) Chronic inflammation is a potent epigenetic modifier by inhibiting the DNMT activity, which leads to passive demethylation during cell division, and activating the Ten‐Eleven Translocation (TET) enzymes, which catalyze the active demethylation of 5mC (Oliveira et al. 2009; Takeshima et al. 2020). This whole process is often mediated by the NF‐κB signaling pathway.

By and large, the ultimate outcome also depends on several context‐related factors, including the bacterial virulence factor, the duration and stage of the inflammation, and the cellular and chromatin context, for instance, the baseline accessibility of the promoter.

## 6 Future Perspectives: Bridging Gaps and Pioneering Approaches

While the previous section outlined currently available clinical applications, we now turn to emerging approaches still in development. Notably, the strategies discussed below require further validation before clinical implementation.

An enduring challenge in this field is distinguishing correlation from causality. There remains controversy over whether periodontitis‐associated bacteria actively promote HNSCCs or are secondary colonizers of a favorable tumor niche (Teles et al. 2020; Pigossi et al. 2025). Methods such as Mendelian randomization (MR) have been used to address this question, with studies providing both supportive and non‐supportive evidence (Wu, Peng et al. 2025; Xiong et al. 2024; Xiao et al. 2025). Notably, stronger evidence comes from animal studies, which suggest a causal link between periodontitis‐related bacteria and cancer incidence. Conversely, human‐based research suggests mainly correlation (Pigossi et al. 2025). While the preclinical evidence suggests that periodontal pathogens actively contribute to HNSCC progression, definitive proof in humans requires prospective interventional studies and carefully controlled longitudinal cohorts. Taken together, these bacteria appear to be active contributors to the oncogenic process, engaging in a complex, bidirectional relationship with the evolving TME.

Clustered regularly interspaced short palindromic repeats (CRISPR) and CRISPR‐associated systems (Cas) consist of DNA sequences that can activate or deactivate specific genetic sequences. CRISPR‐Cas systems are naturally present in periodontal pathogens such as *P. gingivalis* and *T. denticola*, functioning as an adaptive immune response against various microbes (Watanabe et al. 2017). These systems can be repurposed to disrupt specific virulence genes, such as *fadA* in *F. nucleatum* or *gingipains* in *P. gingivalis*, without harming the commensal flora, thereby delivering antimicrobial activity while reducing microbial resistance (Yadalam et al. 2023). For instance, engineered phages armed with CRISPR‐Cas can evade bacterial defenses, such as anti‐CRISPR proteins (Acrs), which *P. gingivalis* uses to circumvent phage predation (Ceballos‐Garzon et al. 2022). Editing the pathogen genome may reduce oncogenic signaling while preserving symbiotic microbes.

Although promising, major translational hurdles still need to be addressed. The TME and periodontal pockets are hypoxic, complicated, and protected by several biofilms and the ECM. Delivering CRISPR‐carrying vectors to a specific site with sufficient concentration is challenging. Moreover, engineered phages are typically highly specific to a narrow range of bacterial strains, whereas pathogenic strains are diverse within the tumor; thus, non‐target strains will survive (Yadalam et al. 2023; Dziedzic et al. 2025; Lino et al. 2018). Notably, hypothetically eliminating a keystone bacterium such as *P. gingivalis* could cause an unpredictable shift in microbial community structure, potentially favoring other opportunistic species. Lastly, as mentioned above, bacteria can evolve to resist CRISPR‐based therapies, potentially rendering them ineffective. In addition, the human immune system neutralizes the foreign‐engineered phage vectors, further rendering them ineffective (Ceballos‐Garzon et al. 2022). Meanwhile, the regulatory pathway, scalable manufacturing, and standardization of this therapy pose significant logistical and economic challenges.

Recently, epigenetic targeting has garnered vast attention. The FDA has approved several epi‐drugs, which are currently in clinical trials. Additionally, combination treatment of epi‐drugs with immune checkpoint inhibitors (ICIs) is under investigation for various malignancies, including HNSCCs. Azacytidine and Decitabine, as DNMT inhibitors, are being evaluated with Nivolumab and Durvalumab, respectively, in clinical trials for HNSCCs (NCT05317000, NCT03019003). Moreover, Vorinostat, as an HDAC inhibitor, in combination with Pembrolizumab, is already under evaluation for HNSCCs and salivary gland cancers (NCT02538510) (Yang et al. 2023). Using epigenetic drugs in HNSCC treatment may reverse bacteria‐induced epigenetic alterations; for instance, the demethylation of the *TLR2* promoter induced by *P. gingivalis* may help restore immune surveillance (Li and Lu 2024).

Oncobiosis is a dysbiotic state in which periodontal pathogens dominate, thus creating a pro‐carcinogenic niche. Computational tools, such as machine learning models, can detect oncobiotic signatures, suggesting microbiome‐targeted therapies to tailor treatment for individuals with HNSCC (Sahin and Sonmezer 2024). For instance, *P. gingivalis* dominance correlates with PD‐L1 upregulation, indicating the shift toward a PD‐L1‐positive HNSCC treatment regime.

## 7 Clinical Implications: From Bench to Bedside

Building on the mechanistic insights presented earlier, this section focuses on immediate clinical applications that can be implemented in current practice, distinguishing them from the more speculative approaches discussed above.

HNSCCs and periodontal diseases share common risk factors, including smoking, alcohol, and immune suppression. Moreover, recent evidence indicates that oral dysbiosis can drive carcinogenesis through chronic inflammation, immune modulation, and metabolic reprogramming. However, oral health has been neglected in oncology (Tezal et al. 2009). Dentists and oncologists should collaborate to manage oral dysbiosis before and after HNSCC treatment. Regular scaling and root planing for high‐risk patients (e.g., smokers, HPV+) with periodontitis may reduce cancer risk. Furthermore, mandatory periodontal screening in oncology clinics for patients with HNSCC may improve disease outcomes.

Notably, a recent meta‐analysis found that higher intratumoral *F. nucleatum* abundance was associated with improved overall and disease‐specific survival in head and neck cancers, but emphasized the limited evidence base and residual heterogeneity (Bayrak et al. 2025). Thus, from a prognostic standpoint, these findings should be interpreted cautiously and validated prospectively.

Repurposing drugs targeting oral dysbiosis, such as antibiotics, probiotics, and antimicrobials, may also enhance treatment of HNSCC patients, particularly when combined with ICIs. Metronidazole can be used with ICIs in patients with *P. gingivalis* or *F. nucleatum*‐positive tumors. However, it can also disrupt commensal bacteria; hence, pharmacokinetic studies are necessary (Kavitha et al. 2025).

Salivary microbiome sequencing, including nanopore and 16S rRNA sequencing, provides a noninvasive strategy for identifying microbiome‐driven HNSCC subtypes and personalizing treatment approaches. For instance, *F. nucleatum*‐enriched HNSCCs show more Wnt/β‐catenin pathway activation, suggesting using Wnt pathway inhibitors, such as LGK974. Additionally, *P. gingivalis* upregulates PD‐L1. Thus, individuals may benefit from anti‐PD‐1 therapies (Ting et al. 2023).

## 8 Conclusion

It has become increasingly evident that oral dysbiosis can enhance the pathogenicity of HNSCCs across all stages. Available data suggest that periodontitis‐associated bacteria, particularly *P. gingivalis* and *F. nucleatum*, may contribute to cancer progression by affecting cell survival, proliferation, apoptosis, and invasion, either directly or indirectly. This underscores their potential as biomarkers and therapeutic targets. Future research should explore microbiome‐targeted therapies, including probiotics, antimicrobial peptides, and CRISPR‐based bacterial modulation. Additionally, studies should assess the efficacy of combining ICIs with microbial‐targeting drugs to overcome resistance. A multidisciplinary collaboration among dentistry, oncology, and microbiome research is crucial to translating these insights into clinical practice. By addressing the silent role of periodontitis in carcinogenesis, we can develop innovative prevention and treatment strategies that ultimately improve outcomes for patients with HNSCC.

## Author Contributions

**Yashmin Afshar:** conceptualization, writing the original draft, visualization, review and editing. **Nima Rezaei:** conceptualization, project administration.

## Funding

The authors have nothing to report.

## Ethics Statement

The authors have nothing to report.

## Consent

The authors have nothing to report.

## Conflicts of Interest

The authors declare no conflicts of interest.

## Data Availability

The authors have nothing to report.

## References

[cre270341-bib-0001] Alaei, S. R. , A. J. King , K. Banani , et al. 2024. “Lipid a Remodeling Modulates Outer Membrane Vesicle Biogenesis by *Porphyromonas gingivalis* .” Journal of Bacteriology 207: e00336‐24. 39660885 10.1128/jb.00336-24 PMC11784228

[cre270341-bib-0002] Ang, K. K. , J. Harris , R. Wheeler , et al. 2010. “Human Papillomavirus and Survival of Patients With Oropharyngeal Cancer.” New England Journal of Medicine 363, no. 1: 24–35. 20530316 10.1056/NEJMoa0912217 PMC2943767

[cre270341-bib-0003] Aral, K. , M. R. Milward , D. Gupta , and P. R. Cooper . 2020. “Effects of *Porphyromonas gingivalis* and *Fusobacterium nucleatum* on Inflammasomes and Their Regulators in H400 Cells.” Molecular Oral Microbiology 35, no. 4: 158–167. 32516848 10.1111/omi.12302

[cre270341-bib-0004] Azevedo, A. M. , L. P. Carvalho Rocha , S. A. de Faria Amormino , et al. 2020. “DNA Methylation Profile of Genes Related to Immune Response in Generalized Periodontitis.” Journal of Periodontal Research 55, no. 3: 426–431. 31943216 10.1111/jre.12726

[cre270341-bib-0005] Barros, S. P. , and S. Offenbacher . 2014. “Modifiable Risk Factors in Periodontal Disease: Epigenetic Regulation of Gene Expression in the Inflammatory Response.” Periodontology 2000 64, no. 1: 95–110. 24320958 10.1111/prd.12000

[cre270341-bib-0006] Barsouk, A. , J. S. Aluru , P. Rawla , K. Saginala , and A. Barsouk . 2023. “Epidemiology, Risk Factors, and Prevention of Head and Neck Squamous Cell Carcinoma.” Medical Sciences 11, no. 2: 42. 37367741 10.3390/medsci11020042 PMC10304137

[cre270341-bib-0007] Bayrak, A. F. , M. E. Arayici , O. Savas , E. Ozgur , A. Ozkutuk , and E. A. Guneri . 2025. “Prognostic Impact of *Fusobacterium nucleatum* in Head and Neck Cancers: A Meta‐Analysis of Critical Oncological Outcomes.” BMC Cancer 26, no. 1: 35. 41318441 10.1186/s12885-025-15404-1 PMC12781759

[cre270341-bib-0008] Benakanakere, M. , M. Abdolhosseini , K. Hosur , L. S. Finoti , and D. F. Kinane . 2015. “TLR2 Promoter Hypermethylation Creates Innate Iimmune Dysbiosis.” Journal of Dental Research 94, no. 1: 183–191. 25389002 10.1177/0022034514557545 PMC4270813

[cre270341-bib-0009] Benakanakere, M. R. , L. Finoti , D. B. Palioto , H. S. Teixeira , and D. F. Kinane . 2019. “Epigenetics, Inflammation, and Periodontal Disease.” Current Oral Health Reports 6, no. 1: 37–46.

[cre270341-bib-0010] Bertani, B. , and N. Ruiz . 2018. “Function and Biogenesis of Lipopolysaccharides.” Ecosal Plus 8, no. 1. 10.1128/ecosalplus.ESP-0001-2018. PMC6091223 30066669

[cre270341-bib-0011] Bostanci, N. , and G. N. Belibasakis . 2012. “ *Porphyromonas gingivalis*: An Invasive and Evasive Opportunistic Oral Pathogen.” FEMS Microbiology Letters 333, no. 1: 1–9. 22530835 10.1111/j.1574-6968.2012.02579.x

[cre270341-bib-0012] Burcher, K. M. , J. T. Burcher , L. Inscore , C. H. Bloomer , C. M. Furdui , and M. Porosnicu . 2022. “A Review of the Role of Oral Microbiome in the Development, Detection, and Management of Head and Neck Squamous Cell Cancers.” Cancers 14, no. 17: 4116. 36077651 10.3390/cancers14174116 PMC9454796

[cre270341-bib-0013] Cai, L. , H. Zhu , Q. Mou , et al. 2024. “Integrative Analysis Reveals Associations Between Oral Microbiota Dysbiosis and Host Genetic and Epigenetic Aberrations in Oral Cavity Squamous Cell Carcinoma.” NPJ Biofilms and Microbiomes 10, no. 1: 39. 38589501 10.1038/s41522-024-00511-x PMC11001959

[cre270341-bib-0014] de Camargo Pereira, G. , G. N. Guimarães , A. C. Planello , et al. 2013. “ *Porphyromonas gingivalis* LPS Stimulation Downregulates DNMT1, DNMT3a, and JMJD3 Gene Expression Levels in Human HaCaT Keratinocytes.” Clinical Oral Investigations 17: 1279–1285. 22875665 10.1007/s00784-012-0816-z

[cre270341-bib-0015] Carvalho‐Filho, P. C. , I. S. Gomes‐Filho , R. Meyer , T. Olczak , M. T. Xavier , and S. C. Trindade . 2016. “Role of *Porphyromonas gingivalis* HmuY in Immunopathogenesis of Chronic Periodontitis.” Mediators of Inflammation 2016, no. 1: 7465852. 27403039 10.1155/2016/7465852 PMC4925967

[cre270341-bib-0016] Ceballos‐Garzon, A. , A. B. Muñoz , J. D. Plata , Z. A. Sanchez‐Quitian , and J. Ramos‐Vivas . 2022. “Phages, Anti‐CRISPR Proteins, and Drug‐Resistant Bacteria: What Do We Know About This Triad?” Pathogens and Disease 80, no. 1: ftac039. 36255384 10.1093/femspd/ftac039

[cre270341-bib-0017] Chang, C. , H. Wang , J. Liu , et al. 2019. “ *Porphyromonas gingivalis* Infection Promoted the Proliferation of Oral Squamous Cell Carcinoma Cells Through the miR‐21/PDCD4/AP‐1 Negative Signaling Pathway.” ACS Infectious Diseases 5, no. 8: 1336–1347. 31243990 10.1021/acsinfecdis.9b00032

[cre270341-bib-0018] Chattopadhyay, I. , M. Verma , and M. Panda . 2019. “Role of Oral Microbiome Signatures in Diagnosis and Prognosis of Oral Cancer.” Technology in Cancer Research & Treatment 18: 1533033819867354. 31370775 10.1177/1533033819867354 PMC6676258

[cre270341-bib-0019] Chaushu, S. , A. Wilensky , C. Gur , et al. 2012. “Direct Recognition of *Fusobacterium nucleatum* by the NK Cell Natural Cytotoxicity Receptor NKp46 Aggravates Periodontal Disease.” PLoS Pathogens 8, no. 3: 1002601. 10.1371/journal.ppat.1002601 PMC3310798 22457623

[cre270341-bib-0020] Chen, Y. L. , A. Yousif , C. H. Chen , et al. 2025. “Chronic ISG15 Exposure Accelerates CD8+ T‐cell Dysfunction While Increasing PD‐1 Blockade Sensitivity in Oral Squamous Cell Carcinoma.” Cancer Immunology Research 13, no. 11: 1829–1844. 40879086 10.1158/2326-6066.CIR-25-0047 PMC12755121

[cre270341-bib-0021] Chen, Y. P. , A. T. C. Chan , Q. T. Le , P. Blanchard , Y. Sun , and J. Ma . 2019. “Nasopharyngeal Carcinoma.” Lancet 394, no. 10192: 64–80. 31178151 10.1016/S0140-6736(19)30956-0

[cre270341-bib-0022] Cheng, R. , W. Liu , R. Zhang , Y. Feng , N. A. Bhowmick , and T. Hu . 2017. “ *Porphyromonas gingivalis*‐Derived Lipopolysaccharide Combines Hypoxia to Induce Caspase‐1 Activation in Periodontitis.” Frontiers in Cellular and Infection Microbiology 7: 474. 29184853 10.3389/fcimb.2017.00474 PMC5694474

[cre270341-bib-0023] Coleman, J. L. , J. L. Benach , and A. W. Karzai . 2021. “Endogenous and Borrowed Proteolytic Activity in the Borrelia.” Microbiology and Molecular Biology Reviews 85, no. 2: 00217–00220. 10.1128/mmbr. PMC8139524 33980587

[cre270341-bib-0024] Da, J. , X. Wang , L. Li , and Y. Xu . 2021. “ *Fusobacterium nucleatum* Promotes Cisplatin‐Resistance and Migration of Oral Squamous Carcinoma Cells by Up‐Regulating Wnt5a‐Mediated NFATc3 Expression.” Tohoku Journal of Experimental Medicine 253, no. 4: 249–259. 33840648 10.1620/tjem.253.249

[cre270341-bib-0025] Ding, P.‐H. , R. P. Darveau , C.‐Y. Wang , and L. Jin . 2017. “3LPS‐Binding Protein and Its Interactions With *P. gingivalis* LPS Modulate Pro‐Inflammatory Response and Toll‐Like Receptor Signaling in Human Oral Keratinocytes.” PLoS One 12, no. 4: e0173223. 28384159 10.1371/journal.pone.0173223 PMC5383028

[cre270341-bib-0026] Doan, N. B. , H. Alhajala , M. M. Al‐Gizawiy , et al. 2017. “Acid Ceramidase and Its Inhibitors: A de Novo Drug Target and a New Class of Drugs for Killing Glioblastoma Cancer Stem Cells With High Efficiency.” Oncotarget 8, no. 68: 112662. 29348854 10.18632/oncotarget.22637 PMC5762539

[cre270341-bib-0027] Dziedzic, A. , R. Kubina , M. Skonieczna , et al. 2025. “CRISPR Genome Editing in Personalized Therapy for Oral and Maxillofacial Diseases: A Scoping Review.” Biomedicines 13, no. 11: 2745. 41301838 10.3390/biomedicines13112745 PMC12650159

[cre270341-bib-0028] Forbes, S. A. , G. Bhamra , and S. Bamford , et al. 2008. “The Catalogue of Somatic Mutations in Cancer (COSMIC).” Current Protocols in Human Genetics 57, no. 1: Unit 10.11. 10.1002/0471142905.hg1011s57 PMC2705836 18428421

[cre270341-bib-0029] Galeano Niño, J. L. , H. Wu , K. D. LaCourse , et al. 2022. “Effect of the Intratumoral Microbiota on Spatial and Cellular Heterogeneity in Cancer.” Nature 611, no. 7937: 810–817. 36385528 10.1038/s41586-022-05435-0 PMC9684076

[cre270341-bib-0030] Garud, N. R. , and K. S. Pollard . 2020. “Population Genetics in the Human Microbiome.” Trends in Genetics: TIG 36, no. 1: 53–67. 31780057 10.1016/j.tig.2019.10.010

[cre270341-bib-0031] Geng, F. , Y. Zhang , Z. Lu , S. Zhang , and Y. Pan . 2020. “ *Fusobacterium nucleatum* Caused DNA Damage and Promoted Cell Proliferation by the Ku70/p53 Pathway in Oral Cancer Cells.” DNA and Cell Biology 39, no. 1: 144–151. 31765243 10.1089/dna.2019.5064 PMC6978777

[cre270341-bib-0032] Gillison, M. L. , W. M. Koch , R. B. Capone , et al. 2000. “Evidence for a Causal Association Between Human Papillomavirus and a Subset of Head and Neck Cancers.” Journal of the National Cancer Institute 92, no. 9: 709–720. 10793107 10.1093/jnci/92.9.709

[cre270341-bib-0033] Golusińska‐Kardach, E. , H. Yadav , S. Jain , M. M. Masternak , and W. Golusiński . 2025. “The Oral Microbiome and Head and Neck Cancer: A Narrative Review.” Cancers 17, no. 17: 2736. 40940833 10.3390/cancers17172736 PMC12427417

[cre270341-bib-0034] Groeger, S. , E. Domann , J. R. Gonzales , T. Chakraborty , and J. Meyle . 2011. “B7‐H1 and B7‐DC Receptors of Oral Squamous Carcinoma Cells Are Upregulated by *Porphyromonas gingivalis* .” Immunobiology 216, no. 12: 1302–1310. 21723642 10.1016/j.imbio.2011.05.005

[cre270341-bib-0035] Gronow, S. , and H. Brade . 2001. “Lipopolysaccharide Biosynthesis: Which Steps Do Bacteria Need to Survive?” Journal of Endotoxin Research 7, no. 1: 3–23. 11521077

[cre270341-bib-0036] Ha, N. H. , B. H. Woo , D. J. Kim , et al. 2015. “Prolonged and Repetitive Exposure to *Porphyromonas gingivalis* Increases Aggressiveness of Oral Cancer Cells by Promoting Acquisition of Cancer Stem Cell Properties.” Tumour Biology 36, no. 12: 9947–9960. 26178482 10.1007/s13277-015-3764-9

[cre270341-bib-0037] Hajishengallis, G. , M. Wang , and S. Liang . 2009. “Induction of Distinct TLR2‐Mediated Proinflammatory and Proadhesive Signaling Pathways in Response to *Porphyromonas gingivalis* Fimbriae.” Journal of Immunology 182, no. 11: 6690–6696. 10.4049/jimmunol.0900524 PMC2685460 19454663

[cre270341-bib-0038] Han, Q. , R. Wang , C. Sun , et al. 2014. “Human Beta‐Defensin‐1 Suppresses Tumor Migration and Invasion and is an Independent Predictor for Survival of Oral Squamous Cell Carcinoma Patients.” PLoS One 9, no. 3: e91867. 24658581 10.1371/journal.pone.0091867 PMC3962354

[cre270341-bib-0039] Han, Y. W. 2015. “ *Fusobacterium nucleatum*: A Commensal‐Turned Pathogen.” Current Opinion in Microbiology 23: 141–147. 25576662 10.1016/j.mib.2014.11.013 PMC4323942

[cre270341-bib-0040] Herath, T. D. , Y. Wang , C. J. Seneviratne , R. P. Darveau , C.‐Y. Wang , and L. Jin . 2013. “The Expression and Regulation of Matrix Metalloproteinase‐3 Is Critically Modulated by *Porphyromonas gingivalis* Lipopolysaccharide With Heterogeneous Lipid A Structures in Human Gingival Fibroblasts.” BMC Microbiology 13: 1–12. 23548063 10.1186/1471-2180-13-73 PMC3623786

[cre270341-bib-0041] Hsueh, C.‐Y. , Q. Huang , and H. Gong , et al. 2022. “A Positive Feed‐Forward Loop Between *Fusobacterium nucleatum* and Ethanol Metabolism Reprogramming Drives Laryngeal Cancer Progression and Metastasis.” iScience 25, no. 2: 103829. 10.1016/j.isci.2022.103829europepmc.org/articles/PMC8851092europepmc.org/articles/PMC8851092?pdf=render. 35198889 PMC8851092

[cre270341-bib-0042] Hsueh, C.‐Y. , H.‐C. Lau , and Q. Huang , et al. 2022. “ *Fusobacterium nucleatum* Impairs DNA Mismatch Repair and Stability in Patients With Squamous Cell Carcinoma of the Head and Neck.” Cancer 128, no. 17: 3170–3184. 35789992 10.1002/cncr.34338

[cre270341-bib-0043] Hussan, H. , S. K. Clinton , K. Roberts , and M. T. Bailey . 2017. “Fusobacterium′s Link to Colorectal Neoplasia Sequenced: A Systematic Review and Future Insights.” World Journal of Gastroenterology 23, no. 48: 8626–8650. 29358871 10.3748/wjg.v23.i48.8626 PMC5752723

[cre270341-bib-0044] Inaba, H. , H. Sugita , M. Kuboniwa , et al. 2014. “ *Porphyromonas gingivalis* Promotes Invasion of Oral Squamous Cell Carcinoma Through Induction of proMMP9 and Its Activation.” Cellular Microbiology 16, no. 1: 131–145. 23991831 10.1111/cmi.12211 PMC3939075

[cre270341-bib-0045] Jurdziński, K. T. , J. Potempa , and A. M. Grabiec . 2020. “Epigenetic Regulation of Inflammation in Periodontitis: Cellular Mechanisms and Therapeutic Potential.” Clinical Epigenetics 12, no. 1: 186. 33256844 10.1186/s13148-020-00982-7 PMC7706209

[cre270341-bib-0046] Kajiwara, K. , S. Takata , T. T. To , et al. 2017. “The Promotion of Nephropathy by *Porphyromonas gingivalis* Lipopolysaccharide via Toll‐Like Receptors.” Diabetology & Metabolic Syndrome 9: 1–13. 29018490 10.1186/s13098-017-0271-8 PMC5610442

[cre270341-bib-0047] Kamarajan, P. , I. Ateia , and J. M. Shin , et al. 2020. “Periodontal Pathogens Promote Cancer Aggressivity via TLR/MyD88 Triggered Activation of Integrin/FAK Signaling That Is Therapeutically Reversible by a Probiotic Bacteriocin.” PLoS Pathogens 16, no. 10: e1008881. 33002094 10.1371/journal.ppat.1008881 PMC7529280

[cre270341-bib-0048] Kavitha, L. , M. Kuzhalmozhi , J. Vijayashree Priyadharsini , A. Arun Kumar , K. M. R. Umadevi , and K. Ranganathan . 2025. “Microbial Signatures in Head and Neck Squamous Cell Carcinoma: An In Silico Study.” Journal of Applied Oral Science 33: e20240392. 39907412 10.1590/1678-7757-2024-0392 PMC11816647

[cre270341-bib-0049] Kirthika, P. , K. K. S. Lloren , V. Jawalagatti , and J. H. Lee . 2023. “Structure, Substrate Specificity and Role of Lon Protease in Bacterial Pathogenesis and Survival.” International Journal of Molecular Sciences 24, no. 4: 3422. 36834832 10.3390/ijms24043422 PMC9961632

[cre270341-bib-0050] Kompuinen, J. , M. Keskin , D. Yilmaz , M. Gürsoy , and U. K. Gürsoy . 2023. “Human β‐Defensins in Diagnosis of Head and Neck Cancers.” Cells 12, no. 6: 830. 36980171 10.3390/cells12060830 PMC10047923

[cre270341-bib-0051] Kwak, S. , C. Wang , and M. Usyk , et al. 2024. “Oral Microbiome and Subsequent Risk of Head and Neck Squamous Cell Cancer.” JAMA Oncology 10, no. 11: 1537–1547. 39325441 10.1001/jamaoncol.2024.4006 PMC11428028

[cre270341-bib-0052] Larsson, L. , P. M. Giraldo‐Osorno , C. Garaicoa‐Pazmino , W. V. Giannobile , and F. Asa'ad . 2025. “DNA and RNA Methylation in Periodontal and Peri‐implant Diseases.” Journal of Dental Research 104, no. 2: 131–139. 39629934 10.1177/00220345241291533 PMC11752639

[cre270341-bib-0053] Lavu, V. , V. Venkatesan , and S. R. Rao . 2015. “The Epigenetic Paradigm in Periodontitis Pathogenesis.” Journal of Indian Society of Periodontology 19, no. 2: 142–149. 26015662 10.4103/0972-124X.145784 PMC4439621

[cre270341-bib-0054] Lawrence, M. S. , C. Sougnez , L. Lichtenstein , et al. 2015. “Comprehensive Genomic Characterization of Head and Neck Squamous Cell Carcinomas.” Nature 517, no. 7536: 576–582. 25631445 10.1038/nature14129 PMC4311405

[cre270341-bib-0055] Leemans, C. R. , P. J. F. Snijders , and R. H. Brakenhoff . 2018. “The Molecular Landscape of Head and Neck Cancer.” Nature Reviews Cancer 18, no. 5: 269–282. 29497144 10.1038/nrc.2018.11

[cre270341-bib-0056] Li, F. , H. Huang , J. Xu , et al. 2023. “ *Fusobacterium nucleatum*‐Triggered Purine Metabolic Reprogramming Drives Tumorigenesis in Head and Neck Carcinoma.” Discover Oncology 14, no. 1: 120. 37393565 10.1007/s12672-023-00727-x PMC10315358

[cre270341-bib-0057] Li, T.‐J. , Y.‐h Hao , Y.‐l Tang , and X.‐h Liang . 2022. “Periodontal Pathogens: A Crucial Link Between Periodontal Diseases and Oral Cancer.” Frontiers in Microbiology 13: 919633. 35847109 10.3389/fmicb.2022.919633 PMC9279119

[cre270341-bib-0058] Li, W. , Z. Zhang , R. Wu , et al. 2025. “ *Fusobacterium nucleatum‐*Derived Outer Membrane Vesicles Promote Immunotherapy Resistance via Changes in Tryptophan Metabolism in Tumour‐Associated Macrophages.” Journal of Extracellular Vesicles 14, no. 4: e70070. 40241230 10.1002/jev2.70070 PMC12003102

[cre270341-bib-0059] Li, Y. , H. Guo , X. Wang , Y. Lu , C. Yang , and P. Yang . 2015. “Coinfection With *Fusobacterium nucleatum* Can Enhance the Attachment and Invasion of *Porphyromonas gingivalis* or Aggregatibacter Actinomycetemcomitans to Human Gingival Epithelial Cells.” Archives of Oral Biology 60, no. 9: 1387–1393. 26143497 10.1016/j.archoralbio.2015.06.017

[cre270341-bib-0060] Li, Y. , and C. Lu . 2024. “Targeting Epigenetic Dysregulations in Head and Neck Squamous Cell Carcinoma.” Journal of Dental Research 104: 220345241297122. 10.1177/00220345241297122 PMC12764359 39698794

[cre270341-bib-0061] Li, Y. , X. Tan , X. Zhao , et al. 2020. “Composition and Function of Oral Microbiota Between Gingival Squamous Cell Carcinoma and Periodontitis.” Oral Oncology 107: 104710. 32371264 10.1016/j.oraloncology.2020.104710

[cre270341-bib-0062] Lino, C. A. , J. C. Harper , J. P. Carney , and J. A. Timlin . 2018. “Delivering CRISPR: A Review of the Challenges and Approaches.” Drug Delivery 25, no. 1: 1234–1257. 29801422 10.1080/10717544.2018.1474964 PMC6058482

[cre270341-bib-0063] Lou, F. , L. Yan , S. Luo , et al. 2025. “Dysbiotic Oral Microbiota‐Derived Kynurenine, Induced by Chronic Restraint Stress, Promotes Head and Neck Squamous Cell Carcinoma by Enhancing CD8(+) T Cell Exhaustion.” Gut 74, no. 6: 935–947. 39904603 10.1136/gutjnl-2024-333479 PMC12229062

[cre270341-bib-0064] Lu, Q. , R. P. Darveau , L. P. Samaranayake , C.‐Y. Wang , and L. Jin . 2009. “Differential Modulation of Human {Beta}‐Defensins Expression in Human Gingival Epithelia by *Porphyromonas gingivalis* Lipopolysaccharide With Tetra‐ and Penta‐Acylated Lipid A Structures.” Innate Immunity 15, no. 6: 325–335. 19675119 10.1177/1753425909104899

[cre270341-bib-0065] Lu, Y. , Z. Zheng , Y. Yuan , et al. 2021. “The Emerging Role of Exosomes in Oral Squamous Cell Carcinoma.” Frontiers in Cell and Developmental Biology 9: 628103. 33718365 10.3389/fcell.2021.628103 PMC7951141

[cre270341-bib-0066] Lynch, M. C. , and H. K. Kuramitsu . 1999. “Role of Superoxide Dismutase Activity in the Physiology of *Porphyromonas gingivalis* .” Infection and Immunity 67, no. 7: 3367–3375. 10377114 10.1128/iai.67.7.3367-3375.1999 PMC116519

[cre270341-bib-0067] Mager, D. L. , A. D. Haffajee , P. M. Devlin , C. M. Norris , M. R. Posner , and J. M. Goodson . 2005. “The Salivary Microbiota as a Diagnostic Indicator of Oral Cancer: A Descriptive, Non‐Randomized Study of Cancer‐Free and Oral Squamous Cell Carcinoma Subjects.” Journal of Translational Medicine 3: 27. 15987522 10.1186/1479-5876-3-27 PMC1226180

[cre270341-bib-0068] Martin, S. J. 2020. “The FEBS Journal in 2020: Open Access and Quality Versus Quantity Publishing.” FEBS Journal 287, no. 1: 4–10. 31904913 10.1111/febs.15191

[cre270341-bib-0069] Miao, D. , V. Godovikova , X. Qian , S. Seshadrinathan , Y. L. Kapila , and J. C. Fenno . 2014. “Treponema Denticola Upregulates MMP‐2 Activation in Periodontal Ligament Cells: Interplay Between Epigenetics and Periodontal Infection.” Archives of Oral Biology 59, no. 10: 1056–1064. 24973519 10.1016/j.archoralbio.2014.06.003 PMC4120104

[cre270341-bib-0070] Monteiro, J. S. , K. Kaushik , J. A. A. de Arruda , et al. 2024. “Fungal Footprints in Oral Cancer: Unveiling the Oral Mycobiome.” Frontiers in Oral Health 5: 1360340. 38550775 10.3389/froh.2024.1360340 PMC10973146

[cre270341-bib-0071] Morrison, A. 2024. Dysbiosis of the Oral Microbiome Increases the Progression of Head and Neck Squamous Cell Carcinoma: University of Kansas.

[cre270341-bib-0072] Muijlwijk, T. , I. H. Nauta , A. van der Lee , et al. 2024. “Hallmarks of a Genomically Distinct Subclass of Head and Neck Cancer.” Nature Communications 15, no. 1: 9060. 10.1038/s41467-024-53390-3 PMC11491468 39428388

[cre270341-bib-0073] Naylor, K. L. 2016. The Role of Outer Membrane Proteins of *Porphyromonas gingivalis* in Host‐Pathogen Interactions: University of Sheffield.

[cre270341-bib-0074] Neuzillet, C. , M. Marchais , S. Vacher , et al. 2021. “Prognostic Value of Intratumoral *Fusobacterium nucleatum* and Association With Immune‐Related Gene Expression in Oral Squamous Cell Carcinoma Patients.” Scientific Reports 11, no. 1: 7870. 33846399 10.1038/s41598-021-86816-9 PMC8041800

[cre270341-bib-0075] Nie, F. , J. Zhang , H. Tian , et al. 2024. “The Role of CXCL2‐Mediated Crosstalk Between Tumor Cells and Macrophages in *Fusobacterium nucleatum*‐Promoted Oral Squamous Cell Carcinoma Progression.” Cell Death & Disease 15, no. 4: 277. 38637499 10.1038/s41419-024-06640-7 PMC11026399

[cre270341-bib-0076] Ohshima, J. , Q. Wang , Z. R. Fitzsimonds , et al. 2019. “ *Streptococcus gordonii* Programs Epithelial Cells to Resist ZEB2 Induction by *Porphyromonas gingivalis* .” Proceedings of the National Academy of Sciences of the United States of America 116, no. 17: 8544–8553. 30971493 10.1073/pnas.1900101116 PMC6486779

[cre270341-bib-0077] Oliveira, N. F. , G. R. Damm , and D. C. Andia , et al. 2009. “DNA Methylation Status of the IL8 Gene Promoter in Oral Cells of Smokers and Non‐Smokers With Chronic Periodontitis.” Journal of Clinical Periodontology 36, no. 9: 719–725. 19659670 10.1111/j.1600-051X.2009.01446.x

[cre270341-bib-0078] Omori, Y. , K. Noguchi , M. Kitamura , et al. 2024. “Bacterial Lipopolysaccharide Induces PD‐L1 Expression and an Invasive Phenotype of Oral Squamous Cell Carcinoma Cells.” Cancers (Basel) 16, no. 2: 343. 38254832 10.3390/cancers16020343 PMC10813992

[cre270341-bib-0079] Orlandi, E. , N. A. Iacovelli , V. Tombolini , et al. 2019. “Potential Role of Microbiome in Oncogenesis, Outcome Prediction and Therapeutic Targeting for Head and Neck Cancer.” Oral Oncology 99: 104453. 31683170 10.1016/j.oraloncology.2019.104453

[cre270341-bib-0080] Park, O.‐J. , Y. Kwon , C. Park , et al. 2020. “ *Streptococcus gordonii*: Pathogenesis and Host Response to Its Cell Wall Components.” Microorganisms 8, no. 12: 1852. 33255499 10.3390/microorganisms8121852 PMC7761167

[cre270341-bib-0081] Peng, R. T. , Y. Sun , X. D. Zhou , et al. 2022. “Treponema Denticola Promotes OSCC Development via the TGF‐β Signaling Pathway.” Journal of Dental Research 101, no. 6: 704–713. 35045750 10.1177/00220345211067401

[cre270341-bib-0082] Pigossi, S. C. , J. A. Oliveira , M. C. de Medeiros , L. F. F. Soares , and N. J. D'Silva . 2025. “Demystifying the Link Between Periodontitis and Oral Cancer: A Systematic Review Integrating Clinical, Pre‐Clinical, and In Vitro Data.” Cancer Metastasis Reviews 44, no. 3: 67. 40924302 10.1007/s10555-025-10285-z PMC12420769

[cre270341-bib-0083] Qin, X. , H. Zi , and X. Zeng . 2022. “Changes in the Global Burden of Untreated Dental Caries From 1990 to 2019: A Systematic Analysis for the Global Burden of Disease Study.” Heliyon 8, no. 9: e10714. 10.1016/j.heliyon.2022.e10714https://europepmc.org/articles/PMC9526157https://europepmc.org/articles/PMC9526157?pdf=render. 36193522 PMC9526157

[cre270341-bib-0084] Qin, Y. , Z. Li , T. Liu , et al. 2024. “ *Prevotella intermedia* Boosts OSCC Progression Through ISG15 Upregulation: A New Target for Intervention.” Journal of Cancer Research and Clinical Oncology 150, no. 4: 206. 38644421 10.1007/s00432-024-05730-5 PMC11033248

[cre270341-bib-0085] Rizzato, C. , J. Torres , E. Kasamatsu , et al. 2019. “Potential Role of Biofilm Formation in the Development of Digestive Tract Cancer With Special Reference to Helicobacter pylori Infection.” Frontiers in Microbiology 10: 846. 31110496 10.3389/fmicb.2019.00846 PMC6501431

[cre270341-bib-0086] Romandini, P. , C. Marruganti , W. G. Romandini , M. Sanz , S. Grandini , and M. Romandini . 2024. “Are Periodontitis and Dental Caries Associated? A Systematic Review With Meta‐Analyses.” Journal of Clinical Periodontology 51, no. 2: 145–157. 38084804 10.1111/jcpe.13910

[cre270341-bib-0087] Rubinstein, M. R. , X. Wang , W. Liu , Y. Hao , G. Cai , and Y. W. Han . 2013. “ *Fusobacterium nucleatum* Promotes Colorectal Carcinogenesis by Modulating E‐Cadherin/β‐Catenin Signaling via Its FadA Adhesin.” Cell Host & Microbe 14, no. 2: 195–206. 23954158 10.1016/j.chom.2013.07.012 PMC3770529

[cre270341-bib-0088] Sahin, T. K. , and M. C. Sonmezer . 2024. “The Role of the Microbiome in Head and Neck Squamous Cell Cancers.” European Archives of Oto‐Rhino‐Laryngology 282: 1–15. 39306588 10.1007/s00405-024-08966-6

[cre270341-bib-0089] Saikia, P. J. , L. Pathak , S. Mitra , and B. Das . 2023. “The Emerging Role of Oral Microbiota in Oral Cancer Initiation, Progression and Stemness.” Frontiers in Immunology 14: 1198269. 37954619 10.3389/fimmu.2023.1198269 PMC10639169

[cre270341-bib-0090] Salman, M. , P. Sharma , M. Kumar , A. S. Ethayathulla , and P. Kaur . 2023. “Targeting Novel Sites in DNA Gyrase for Development of Anti‐Microbials.” Briefings in Functional Genomics 22, no. 2: 180–194. 36064602 10.1093/bfgp/elac029

[cre270341-bib-0091] Schmidt, B. L. , J. Kuczynski , A. Bhattacharya , et al. 2014. “Changes in Abundance of Oral Microbiota Associated With Oral Cancer.” PLoS One 9, no. 6: e98741. 24887397 10.1371/journal.pone.0098741 PMC4041887

[cre270341-bib-0092] Schulz, S. , U. D. Immel , L. Just , H.‐G. Schaller , C. Gläser , and S. Reichert . 2016. “Epigenetic Characteristics in Inflammatory Candidate Genes in Aggressive Periodontitis.” Human Immunology 77, no. 1: 71–75. 26472015 10.1016/j.humimm.2015.10.007

[cre270341-bib-0093] Selvaraj, A. , G. McManus , C. M. Healy , and G. P. Moran . 2024. “ *Fusobacterium nucleatum* Induces Invasive Growth and Angiogenic Responses in Malignant Oral Keratinocytes That Are Cell Line‐ and Bacterial Strain‐Specific.” Frontiers in Cellular and Infection Microbiology 14: 1417946. 39286811 10.3389/fcimb.2024.1417946 PMC11402903

[cre270341-bib-0094] Shin, Y. J. , H. W. Choung , J. H. Lee , I. C. Rhyu , and H. D. Kim . 2019. “Association of Periodontitis With Oral Cancer: A Case‐Control Study.” Journal of Dental Research 98, no. 5: 526–533. 30779879 10.1177/0022034519827565

[cre270341-bib-0095] Smeets, S. J. , R. H. Brakenhoff , B. Ylstra , et al. 2009. “Genetic Classification of Oral and Oropharyngeal Carcinomas Identifies Subgroups With a Different Prognosis.” Cellular Oncology 31, no. 4: 291–300. 19633365 10.3233/CLO-2009-0471 PMC4618915

[cre270341-bib-0096] Sobhani, N. , A. D'angelo , F. G. Kugeratski , et al. 2025. “Immune Biomarkers for Head and Neck Cancer.” Cancer Immunology, Immunotherapy: CII 75, no. 1: 6. 41410866 10.1007/s00262-025-04233-7 PMC12715102

[cre270341-bib-0097] Spratt, D. A. , J. Greenman , and A. G. Schaffer . 1995. “ *Capnocytophaga gingivalis* Aminopeptidase: A Potential Virulence Factor.” Microbiology 141, no. Pt 12: 3087–3093. 8574402 10.1099/13500872-141-12-3087

[cre270341-bib-0098] Starska‐Kowarska, K. 2025. “The Role of *Porphyromonas gingivalis* in Oral Carcinogenesis and Progression by Remodelling the Tumour Microenvironment: A Narrative Review.” Cancers (Basel) 17, no. 21: 3478. 41228271 10.3390/cancers17213478 PMC12607439

[cre270341-bib-0099] Steimle, A. , I. B. Autenrieth , and J.‐S. Frick . 2016. “Structure and Function: Lipid A Modifications in Commensals and Pathogens.” International Journal of Medical Microbiology: IJMM 306, no. 5: 290–301. 27009633 10.1016/j.ijmm.2016.03.001

[cre270341-bib-0100] Strati, A. , C. Adamopoulos , I. Kotsantis , A. Psyrri , E. Lianidou , and A. G. Papavassiliou . 2025. “Targeting the PD‐1/PD‐L1 Signaling Pathway for Cancer Therapy: Focus on Biomarkers.” International Journal of Molecular Sciences 26, no. 3: 1235. 39941003 10.3390/ijms26031235 PMC11818137

[cre270341-bib-0101] Sukmana, B. I. , R. O. Saleh , M. A. Najim , et al. 2024. “Oral Microbiota and Oral Squamous Cell Carcinoma: A Review of Their Relation and Carcinogenic Mechanisms.” Frontiers in Oncology 14: 1319777. 38375155 10.3389/fonc.2024.1319777 PMC10876296

[cre270341-bib-0102] Sun, J. , Q. Tang , S. Yu , et al. 2023. “ *F. nucleatum* Facilitates Oral Squamous Cell Carcinoma Progression via GLUT1‐Driven Lactate Production.” EBioMedicine 88: 104444. 36709580 10.1016/j.ebiom.2023.104444 PMC9900488

[cre270341-bib-0103] Swain Ewald, H. A. , and P. W. Ewald . 2020. “Integrating the Microbiome Into the Barrier Theory of Cancer.” Evolutionary Applications 13, no. 7: 1701–1707.

[cre270341-bib-0104] Sztukowska, M. N. , A. Ojo , and S. Ahmed , et al. 2016. “ *Porphyromonas gingivalis* Initiates a Mesenchymal‐Like Transition Through ZEB1 in Gingival Epithelial Cells.” Cellular Microbiology 18, no. 6: 844–858. 26639759 10.1111/cmi.12554 PMC5135094

[cre270341-bib-0105] Takeshima, H. , T. Niwa , S. Yamashita , et al. 2020. “TET Repression and Increased DNMT Activity Synergistically Induce Aberrant DNA Methylation.” Journal of Clinical Investigation 130, no. 10: 5370–5379. 32663196 10.1172/JCI124070 PMC7524486

[cre270341-bib-0106] Tan, Y. , and J. C. Kagan . 2014. “A Cross‐Disciplinary Perspective on the Innate Immune Responses to Bacterial Lipopolysaccharide.” Molecular Cell 54, no. 2: 212–223. 24766885 10.1016/j.molcel.2014.03.012 PMC4096783

[cre270341-bib-0107] Teles, F. R. F. , F. Alawi , R. M. Castilho , and Y. Wang . 2020. “Association or Causation? Exploring the Oral Microbiome and Cancer Links.” Journal of Dental Research 99, no. 13: 1411–1424. 32811287 10.1177/0022034520945242 PMC7684840

[cre270341-bib-0108] Tezal, M. , M. A. Sullivan , A. Hyland , et al. 2009. “Chronic Periodontitis and the Incidence of Head and Neck Squamous Cell Carcinoma.” Cancer Epidemiology, Biomarkers & Prevention 18, no. 9: 2406–2412. 10.1158/1055-9965.EPI-09-0334 19745222

[cre270341-bib-0109] Ting, H. S. L. , Z. Chen , and J. Y. K. Chan . 2023. “Systematic Review on Oral Microbial Dysbiosis and Its Clinical Associations With Head and Neck Squamous Cell Carcinoma.” Head & Neck 45, no. 8: 2120–2135. 37249085 10.1002/hed.27422

[cre270341-bib-0110] Trent, M. S. , C. M. Stead , A. X. Tran , and J. V. Hankins . 2006. “Diversity of Endotoxin and Its Impact on Pathogenesis.” Journal of Endotoxin Research 12, no. 4: 205–223. 16953973 10.1179/096805106X118825

[cre270341-bib-0111] Uitto, V. J. , D. Baillie , Q. Wu , et al. 2005. “ *Fusobacterium nucleatum* Increases Collagenase 3 Production and Migration of Epithelial Cells.” Infection and Immunity 73, no. 2: 1171–1179. 15664960 10.1128/IAI.73.2.1171-1179.2005 PMC547012

[cre270341-bib-0112] Wang, B. , J. Deng , V. Donati , et al. 2024. “The Roles and Interactions of *Porphyromonas gingivalis* and *Fusobacterium nucleatum* in Oral and Gastrointestinal Carcinogenesis: A Narrative Review.” Pathogens 13, no. 1: 93. 38276166 10.3390/pathogens13010093 PMC10820765

[cre270341-bib-0113] Wang, X. , and P. J. Quinn . 2010. “Endotoxins: Lipopolysaccharides of Gram‐Negative Bacteria.” Sub‐Cellular Biochemistry 53: 3–25. 20593260 10.1007/978-90-481-9078-2_1

[cre270341-bib-0114] Watanabe, T. , M. Shibasaki , F. Maruyama , T. Sekizaki , and I. Nakagawa . 2017. “Investigation of Potential Targets of Porphyromonas CRISPRs Among the Genomes of Porphyromonas Species.” PLoS One 12, no. 8: e0183752. 28837670 10.1371/journal.pone.0183752 PMC5570325

[cre270341-bib-0115] Wu, J. S. , M. Zheng , M. Zhang , et al. 2018. “ *Porphyromonas gingivalis* Promotes 4‐Nitroquinoline‐1‐Oxide‐Induced Oral Carcinogenesis With an Alteration of Fatty Acid Metabolism.” Frontiers in Microbiology 9: 2081. 30233549 10.3389/fmicb.2018.02081 PMC6131559

[cre270341-bib-0116] Wu, R. , F. Shao , and S. B. Koh , et al. 2025. “The ISGylation Tapestry in Cancer: Weaving Phenotypic Plasticity Through Multidimensional Regulatory Looms.” Cellular & Molecular Biology Letters 30, no. 1: 132. 41193953 10.1186/s11658-025-00815-6 PMC12590619

[cre270341-bib-0117] Wu, Z. , Q. Peng , and Z. Ren , et al. 2025. “Causal Association Between Oral Microbiota and Oral Cancer: A Mendelian Randomization Study.” Scientific Reports 15, no. 1: 19797. 40473751 10.1038/s41598-025-05553-5 PMC12141564

[cre270341-bib-0118] Xiao, T. , G. Hu , T. Li , X. Zhu , H. Wang , and Z. Zhu . 2025. “Causal Association Between Periodontitis and Oral Cancer: A Two‐Sample Mendelian Randomization Study.” Discover Oncology 16, no. 1: 964. 40445454 10.1007/s12672-025-02528-w PMC12125425

[cre270341-bib-0119] Xiong, J. , H. Liu , C. Li , Y. Li , and J. Feng . 2024. “Linking Periodontitis With 20 Cancers, Emphasis on Oropharyngeal Cancer: A Mendelian Randomization Analysis.” Scientific Reports 14, no. 1: 12511. 38822160 10.1038/s41598-024-63447-4 PMC11143368

[cre270341-bib-0120] Yadalam, P. K. , D. Arumuganainar , R. V. Anegundi , et al. 2023. “CRISPR‐Cas‐Based Adaptive Immunity Mediates Phage Resistance in Periodontal Red Complex Pathogens.” Microorganisms 11, no. 8: 2060. 37630620 10.3390/microorganisms11082060 PMC10459013

[cre270341-bib-0121] Yamada, C. , A. Ho , A. Nusbaum , et al. 2023. “Inhibitory Effect of *Porphyromonas gingivalis*‐Derived Phosphoethanolamine Dihydroceramide on Acid Ceramidase Expression in Oral Squamous Cells.” Journal of Cellular and Molecular Medicine 27, no. 9: 1290–1295. 37016912 10.1111/jcmm.17722 PMC10148054

[cre270341-bib-0122] Yamamoto, Y. , T. Kamiya , M. Yano , et al. 2023. “Oral Microbial Profile Analysis in Patients With Oral and Pharyngeal Cancer Reveals That Tumoral *Fusobacterium nucleatum* Promotes Oral Cancer Progression by Activating YAP.” Microorganisms 11, no. 12: 2957. 38138101 10.3390/microorganisms11122957 PMC10746018

[cre270341-bib-0123] Yang, J. , J. Xu , W. Wang , B. Zhang , X. Yu , and S. Shi . 2023. “Epigenetic Regulation in the Tumor Microenvironment: Molecular Mechanisms and Therapeutic Targets.” Signal Transduction and Targeted Therapy 8, no. 1: 210. 37217462 10.1038/s41392-023-01480-x PMC10203321

[cre270341-bib-0124] Yao, L. , C. Jermanus , B. Barbetta , et al. 2010. “ *Porphyromonas gingivalis* Infection Sequesters Pro‐Apoptotic Bad Through Akt in Primary Gingival Epithelial Cells.” Molecular Oral Microbiology 25, no. 2: 89–101. 20331797 10.1111/j.2041-1014.2010.00569.x PMC2894563

[cre270341-bib-0125] Yao, Y. , X. Shen , M. Zhou , and B. Tang . 2021. “Periodontal Pathogens Promote Oral Squamous Cell Carcinoma by Regulating ATR and NLRP3 Inflammasome.” Frontiers in Oncology 11: 722797. 34660289 10.3389/fonc.2021.722797 PMC8514820

[cre270341-bib-0126] Ye, C. , X. Liu , Z. Liu , et al. 2024. “ *Fusobacterium nucleatum* in Tumors: From Tumorigenesis to Tumor Metastasis and Tumor Resistance.” Cancer Biology & Therapy 25, no. 1: 2306676. 38289287 10.1080/15384047.2024.2306676 PMC10829845

[cre270341-bib-0127] Yilmaz, O. , L. Yao , K. Maeda , et al. 2008. “ATP Scavenging by the Intracellular Pathogen *Porphyromonas gingivalis* Inhibits P2X7‐Mediated Host‐Cell Apoptosis.” Cellular Microbiology 10, no. 4: 863–875. 18005240 10.1111/j.1462-5822.2007.01089.x PMC2637656

[cre270341-bib-0128] Yin, L. , and W. O. Chung . 2011. “Epigenetic Regulation of Human β‐Defensin 2 and CC Chemokine Ligand 20 Expression in Gingival Epithelial Cells in Response to Oral Bacteria.” Mucosal Immunology 4, no. 4: 409–419. 21248725 10.1038/mi.2010.83 PMC3118861

[cre270341-bib-0129] Yuan, K. , S. Xu , G. Liu , et al. 2024. “ *Porphyromonas gingivalis* Promotes Oral Squamous Cell Carcinoma Progression by Modulating Autophagy.” Oral Diseases 31: 492–502. 39435608 10.1111/odi.15157

[cre270341-bib-0130] Zang, W. , F. Geng , J. Liu , et al. 2025. “ *Porphyromonas gingivalis* Potentiates Stem‐Like Properties of Oral Squamous Cell Carcinoma by Modulating SCD1‐Dependent Lipid Synthesis via NOD1/KLF5 Axis.” International Journal of Oral Science 17, no. 1: 15. 40016182 10.1038/s41368-024-00342-8 PMC11868650

[cre270341-bib-0131] Zhang, S. , S. P. Barros , A. J. Moretti , et al. 2013. “Epigenetic Regulation of TNFA Expression in Periodontal Disease.” Journal of Periodontology 84, no. 11: 1606–1616. 23368949 10.1902/jop.2013.120294 PMC3986590

[cre270341-bib-0132] Zhang, S. , S. P. Barros , M. D. Niculescu , A. J. Moretti , J. S. Preisser , and S. Offenbacher . 2010. “Alteration of PTGS2 Promoter Methylation in Chronic Periodontitis.” Journal of Dental Research 89, no. 2: 133–137. 20042743 10.1177/0022034509356512 PMC3065122

[cre270341-bib-0133] Zhang, S. , S. Cai , and Y. Ma . 2018. “Association Between *Fusobacterium nucleatum* and Colorectal Cancer: Progress and Future Directions.” Journal of Cancer 9, no. 9: 1652–1659. 29760804 10.7150/jca.24048 PMC5950595

[cre270341-bib-0134] Zhang, S. , C. Li , J. Liu , et al. 2020. “ *Fusobacterium nucleatum* Promotes Epithelial‐Mesenchymal Transition Through Regulation of the lncRNA MIR4435‐2HG/miR‐296‐5p/Akt2/SNAI1 Signaling Pathway.” FEBS Journal 287, no. 18: 4032–4047. 31997506 10.1111/febs.15233 PMC7540502

[cre270341-bib-0135] Zhang, Z. , S. Liu , S. Zhang , et al. 2022. “ *Porphyromonas gingivalis* Outer Membrane Vesicles Inhibit the Invasion of *Fusobacterium nucleatum* Into Oral Epithelial Cells by Downregulating FadA and FomA.” Journal of Periodontology 93, no. 4: 515–525. 34458990 10.1002/JPER.21-0144 PMC9415117

[cre270341-bib-0136] Zhou, Q. , S. E. Leeman , and S. Amar . 2011. “Signaling Mechanisms in the Restoration of Impaired Immune Function Due to Diet‐Induced Obesity.” Proceedings of the National Academy of Sciences of the United States of America 108, no. 7: 2867–2872. 21282635 10.1073/pnas.1019270108 PMC3041076

[cre270341-bib-0137] Zhou, X. , X. Liu , J. Li , R. M. Aprecio , W. Zhang , and Y. Li . 2015. “Real‐Time PCR Quantification of Six Periodontal Pathogens in Saliva Samples From Healthy Young Adults.” Clinical Oral Investigations 19, no. 4: 937–946. 25217278 10.1007/s00784-014-1316-0

[cre270341-bib-0138] Zhou, Y. , Y. Qin , J. Ma , et al. 2024. “Heat‐Killed *Prevotella intermedia* Promotes the Progression of Oral Squamous Cell Carcinoma by Inhibiting the Expression of Tumor Suppressors and Affecting the Tumor Microenvironment.” Experimental Hematology & Oncology 13, no. 1: 33. 38515216 10.1186/s40164-024-00500-y PMC10956211

[cre270341-bib-0139] Zhu, W. , W. Shen , J. Wang , et al. 2024. “ *Capnocytophaga gingivalis* Is a Potential Tumor Promotor in Oral Cancer.” Oral Diseases 30, no. 2: 353–362. 36093607 10.1111/odi.14376

[cre270341-bib-0140] Zhu, X. X. , X. J. Yang , Y. L. Chao , et al. 2017. “The Potential Effect of Oral Microbiota in the Prediction of Mucositis During Radiotherapy for Nasopharyngeal Carcinoma.” EBioMedicine 18: 23–31. 28216066 10.1016/j.ebiom.2017.02.002 PMC5405060

